# Intercrypt sentinel macrophages tune antibacterial NF-κB responses in gut epithelial cells via TNF

**DOI:** 10.1084/jem.20210862

**Published:** 2021-09-16

**Authors:** Annika Hausmann, Boas Felmy, Leo Kunz, Sanne Kroon, Dorothée Lisa Berthold, Giverny Ganz, Ioana Sandu, Toshihiro Nakamura, Nathan Sébastien Zangger, Yang Zhang, Tamas Dolowschiak, Stefan Alexander Fattinger, Markus Furter, Anna Angelika Müller-Hauser, Manja Barthel, Katerina Vlantis, Laurens Wachsmuth, Jan Kisielow, Luigi Tortola, Danijela Heide, Mathias Heikenwälder, Annette Oxenius, Manfred Kopf, Timm Schroeder, Manolis Pasparakis, Mikael Erik Sellin, Wolf-Dietrich Hardt

**Affiliations:** 1 Institute of Microbiology, Department of Biology, Eidgenössische Technische Hochschule Zurich, Zurich, Switzerland; 2 Department of Biosystems Science and Engineering, Eidgenössische Technische Hochschule Zurich, Basel, Switzerland; 3 Science for Life Laboratory, Department of Medical Biochemistry and Microbiology, Uppsala University, Uppsala, Sweden; 4 Institute for Genetics, Cologne Excellence Cluster on Cellular Stress Responses in Aging-Associated Diseases, University of Cologne, Cologne, Germany; 5 Institute of Molecular Health Sciences, Department of Biology, Eidgenössische Technische Hochschule Zurich, Zurich, Switzerland; 6 Division of Chronic Inflammation and Cancer, German Cancer Research Center, Heidelberg, Germany

## Abstract

Intestinal epithelial cell (IEC) NF-κB signaling regulates the balance between mucosal homeostasis and inflammation. It is not fully understood which signals tune this balance and how bacterial exposure elicits the process. Pure LPS induces epithelial NF-κB activation in vivo. However, we found that in mice, IECs do not respond directly to LPS. Instead, tissue-resident lamina propria intercrypt macrophages sense LPS via TLR4 and rapidly secrete TNF to elicit epithelial NF-κB signaling in their immediate neighborhood. This response pattern is relevant also during oral enteropathogen infection. The macrophage–TNF–IEC axis avoids responses to luminal microbiota LPS but enables crypt- or tissue-scale epithelial NF-κB responses in proportion to the microbial threat. Thereby, intercrypt macrophages fulfill important sentinel functions as first responders to Gram-negative microbes breaching the epithelial barrier. The tunability of this crypt response allows the induction of defense mechanisms at an appropriate scale according to the localization and intensity of microbial triggers.

## Teaser

Hausmann et al. describe a sentinel intercrypt macrophage population in the intestine, which detects Gram-negative microbes breaching the epithelial barrier and elicits epithelial NF-κB signaling in neighboring crypts. The tunability of this crypt-scale response allows the induction of appropriately scaled defenses according to the localization and intensity of microbial triggers.

## Graphical Abstract

**Figure:**
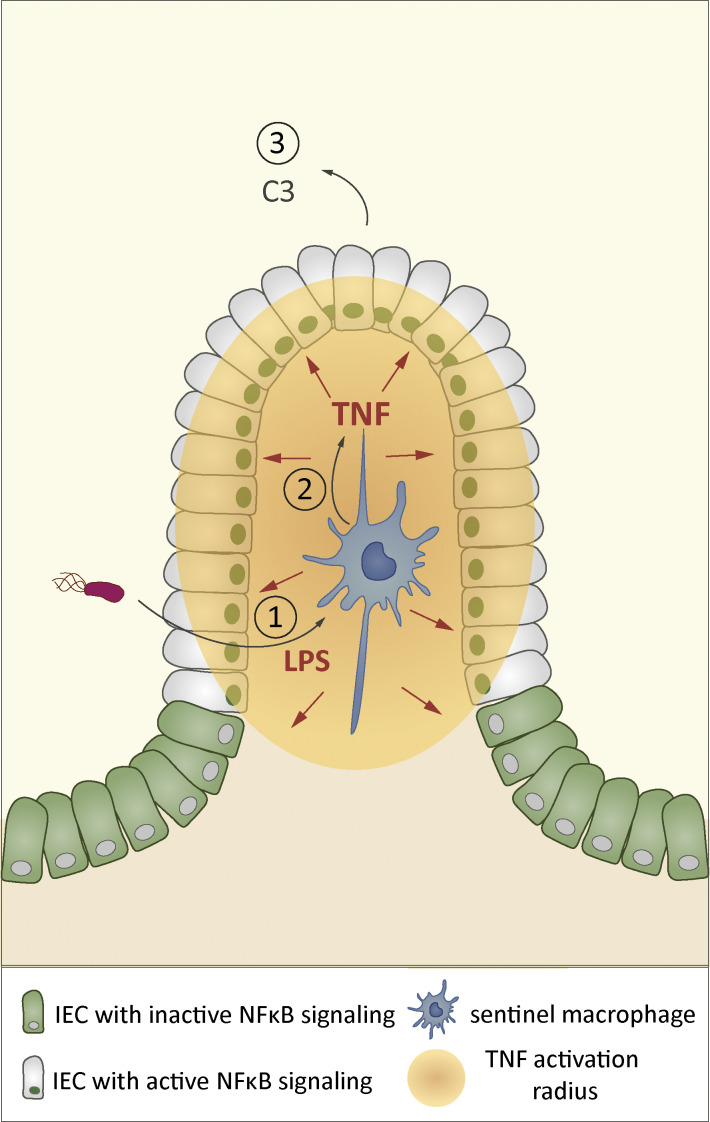

## Introduction

The mucosal immune system maintains host–microbiota homeostasis and defends against pathogen infections. This is a challenging task, as symbionts and pathogens share common microbe-associated molecular patterns, which are recognized by pattern recognition receptors (PRRs; Hausmann and Hardt, 2019). These features make it difficult to distinguish commensals from pathogenic bacteria. Different cell types in the intestinal mucosa act in synergy to achieve this demanding task. Intestinal epithelial cells (IECs) form a physical barrier between the intestinal lumen, containing dense microbial communities, and the sterile host tissue compartment (Hausmann and Hardt, 2019; Johansson and Hansson, 2016). Besides shielding lamina propria (LP) immune cells from luminal microbes, IECs possess sensor and defense effector functions contributing actively to host defense (Birchenough et al., 2016; Cunliffe and Mahida, 2004; Fattinger et al., 2021; Hausmann et al., 2020a; Knodler et al., 2010; Kreibich et al., 2015; Rauch et al., 2017; Sellin et al., 2014; Samperio Ventayol et al., 2021). Immune cell–IEC crosstalk can integrate tissue level signals and induce appropriate responses. Herein, intestinal macrophages are central coordinators of intestinal homeostasis. Their high phagocytic activity and involvement in pathogen defense, inflammation, and tissue repair place them at the nexus of this crosstalk (Arnold et al., 2016; Bain and Schridde, 2018; Bernshtein et al., 2019; Chikina et al., 2020; Corbin et al., 2020; Joeris et al., 2017; Morita et al., 2019; Smith et al., 2011).

PRRs allow a fast detection of microbes. TLR4 and 5 sense bacterial microbe-associated molecular patterns and are widely expressed in mammals (Fitzgerald and Kagan, 2020; Nie et al., 2018). Both immune cells and IECs express TLR5, which detects flagella (Allaire et al., 2021; Fulde et al., 2018; Hayashi et al., 2001; Yang and Yan, 2017). Similarly, immune cell expression of TLR4, which recognizes bacterial LPS (Poltorak et al., 1998), is well described (Medzhitov and Janeway, 2000a). By contrast, its expression by IECs remains controversial. Several studies report TLR4 expression by IECs (Cario et al., 2000; Hornef et al., 2002, 2003; Price et al., 2018; Wang et al., 2010). Contaminations by LP cells in isolated primary IECs, the use of epithelial cell lines that incompletely recapitulate primary IEC expression (Hausmann et al., 2020b), regional and temporal differences in TLR expression (Kayisoglu et al., 2021; Lotz et al., 2006), and the lack of reliable antibodies against TLR4 (Price et al., 2018), however, make these analyses challenging. Adding to this, a number of studies that describe IEC TLR4 expression report little to no functionality of this receptor with regard to LPS sensing (Günther et al., 2015; Kayisoglu et al., 2021; Lotz et al., 2006; Price et al., 2018). Therefore, the functional relevance of IEC TLR4 is still debated, particularly in the intact mucosa.

NF-κB transcription factors integrate numerous signals and drive immune defense. TLR signaling via MyD88 and Ticam1 (Trif; Akira and Hoshino, 2003; Fitzgerald et al., 2003), but also TNF and IL-1 cytokine receptor engagement (Adachi et al., 1998; Hayden and Ghosh, 2014; Muzio et al., 1997) activate the NF-κB pathway. The exact contributions of these stimuli and how they mediate NF-κB signaling across the variety of cell types found in the intestinal mucosa remain incompletely understood. NF-κB transcription factor expression is ubiquitous. Inhibitory regulators sequester them in the cytosol of resting cells (Hayden and Ghosh, 2008). Upon activation, these transcription factors shuttle to the nucleus to induce gene transcription (Adachi et al., 1998; Wang et al., 2001). Epithelial NF-κB signaling maintains intestinal homeostasis by regulating proliferation, survival, and apoptosis of IECs (Guma et al., 2011; Liu et al., 2017; Vlantis et al., 2016; Wullaert et al., 2011). In line with these complex functions, a delicate balance in epithelial NF-κB signaling is crucial. Both inactivation and hyperactivation of this pathway predispose to intestinal inflammation (Dheer et al., 2016; Guma et al., 2011; Vereecke et al., 2010, 2014; Vlantis et al., 2011, 2016; Rogler et al., 1998; Zhang et al., 2006). Hence, epithelial NF-κB can tip the mucosal tissue between homeostasis and inflammation in a fine-tuned manner. To date, it remains unclear what combination of signals and cell types ensures an appropriately balanced NF-κB response in the mucosa upon microbial insult.

We here deciphered how IEC NF-κB signaling is elicited upon exposure to bacterial LPS. In the murine gut, IECs do not directly respond to extracellular LPS. Instead, TNF-producing tissue resident macrophages in intercrypt regions of the LP specifically trigger epithelial NF-κB signaling, inducing a multifaceted, localized, and tunable defense.

## Results

### TLR4^+^ radiosensitive cells induce IEC NF-κB activation upon LPS exposure

To analyze NF-κB signaling dynamics in the gut mucosa, we used p65^GFP-FL^ mice, where the *p65* gene is replaced by a gene encoding a fusion protein of the NF-κB transcription factor p65 and GFP (De Lorenzi et al., 2009). This fusion protein allows real-time assessment of the NF-κB activation status within a cell by monitoring the subcellular localization of the tagged p65 protein. Under homeostatic conditions, p65 resides in the cytosol, while nuclear translocation (and subsequent recycling) occurs upon activation of NF-κB signaling (Adachi et al., 1998).

**Figure 1.**
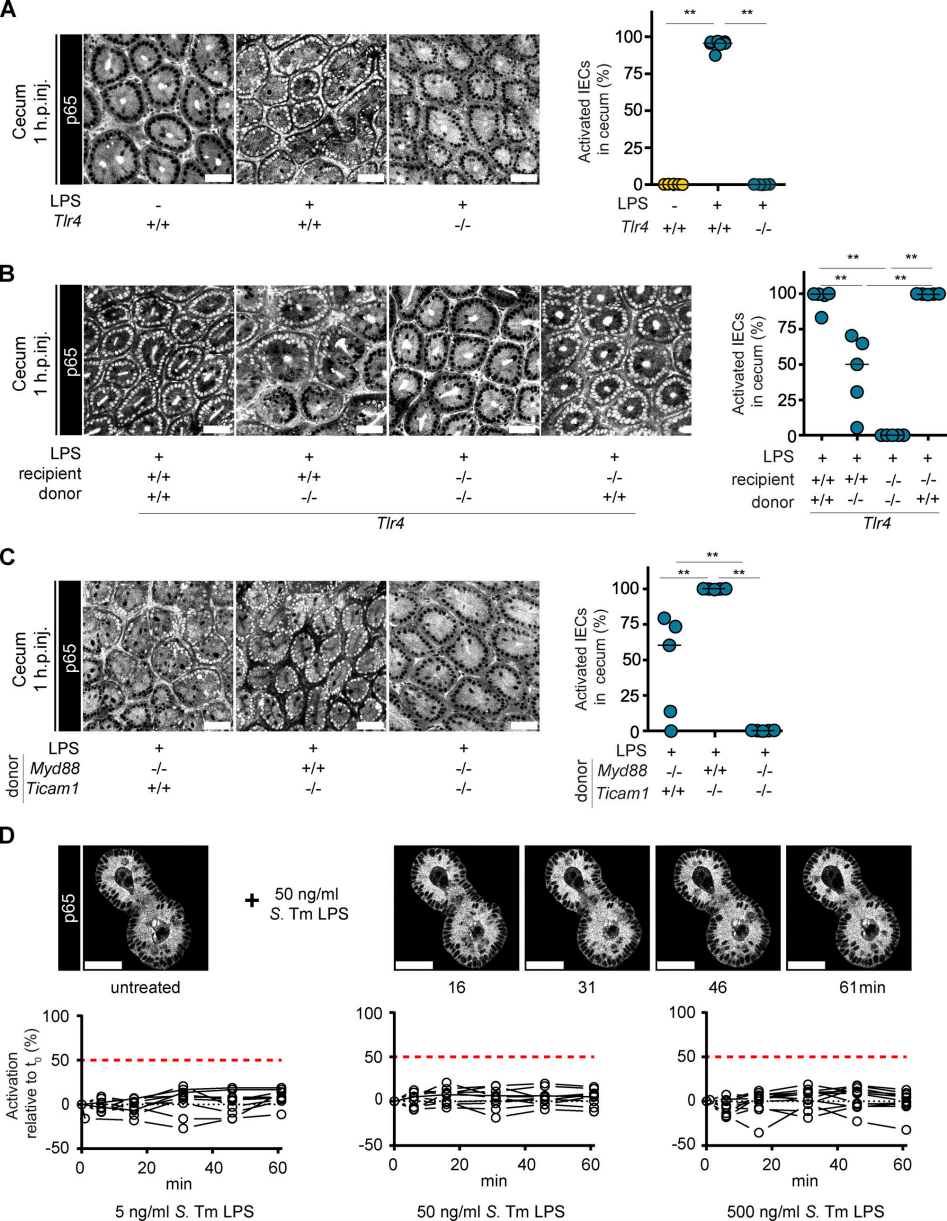
**TLR4*^+^* immune cells induce epithelial NF-κB signaling in the cecal mucosa upon LPS exposure.** Mice were i.v. injected with LPS. Cecal explants were imaged at 1 h.p.inj. by two-photon microscopy. **(A–C)** Representative images and quantification of epithelial NF-κB activation in the indicated mice (A, *n* = 4–7) or BMCs (B, *n* = 5; C, *n* = 5). Each circle represents one mouse. Black line: median. **, P ≤ 0.01 by Mann–Whitney *U* test. **(D)** Small intestinal organoids were treated with 5, 50, or 500 ng/ml LPS and imaged for ∼1 h. Representative images of one organoid over time (top) and quantification of NF-κB activation (bottom; relative change). Each circle represents one organoid at the given time (minutes after start of the treatment, *n* = 7). Lines connect data points from the same organoid. Red dashed line: 50% activation threshold. Black dotted line: no change. Scale bars: 50 µm. Combined data of two (C and D), three (A), or four (B) independent experiments.

**Figure S1.**
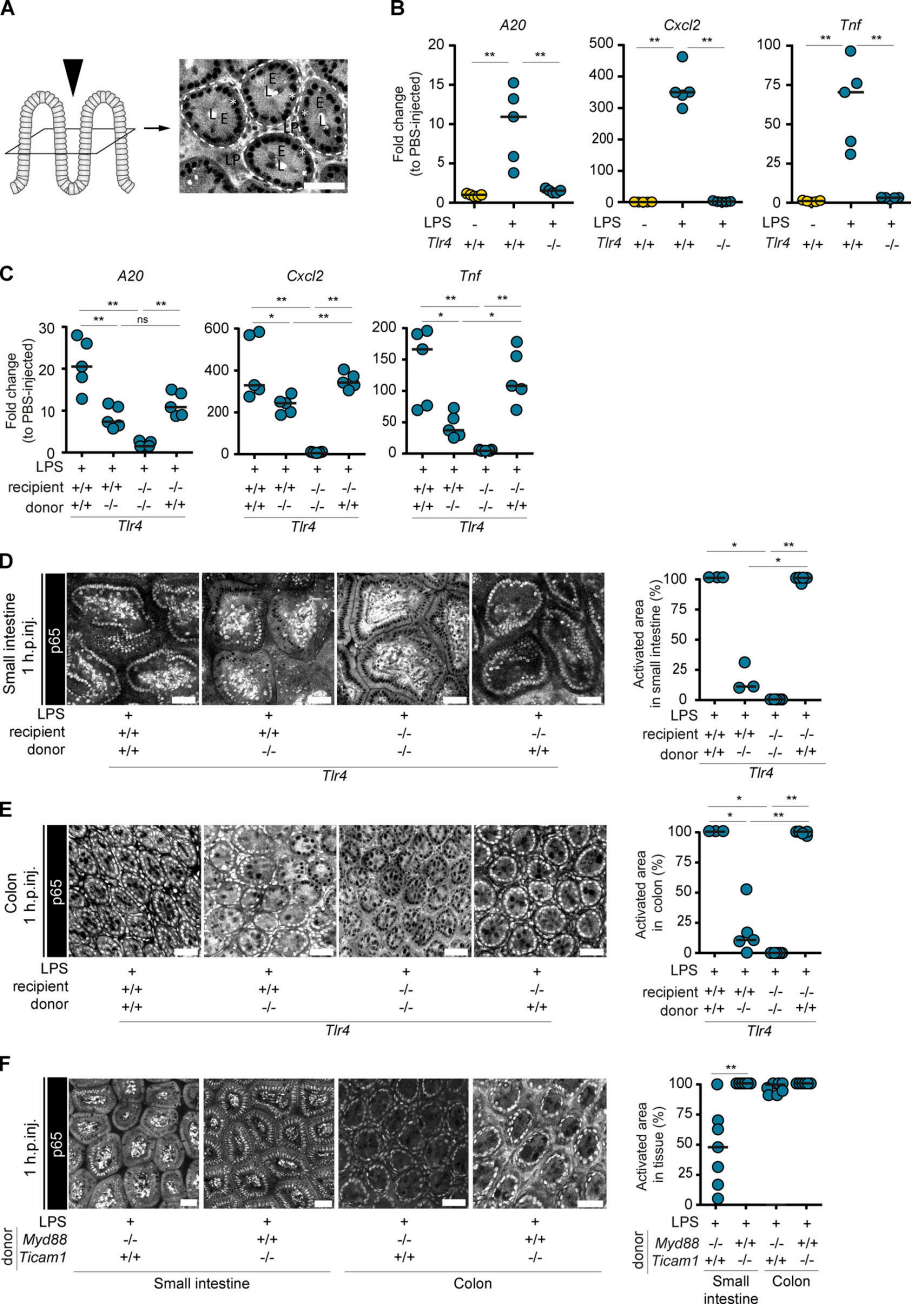
**TLR4^+^ immune cells induce epithelial NF-κB activation in the small intestine and colon. (A)** Schematic drawing of the two-photon imaging (left). The intestinal mucosa is imaged from the luminal side (black arrowhead), resulting in images in horizontal plane of the mucosa (right; part of the image shown in Fig. 1 A). White dashed line/E, epithelium; L, lumen; white asterisks, epithelial nuclei. **(B)** Fold changes in expression of *A20*, *Cxcl2*, and *Tnf* in the cecal mucosa of mice depicted in Fig. 1 A (*n* = 5). **(C)** Fold changes in expression of *A20*, *Cxcl2*, and *Tnf* in the cecal mucosa of mice depicted in Fig. 1 B in comparison to PBS-injected mice in Fig. 1 A (*n* = 5). **(D and E)** Two-photon microscopy images and quantification of epithelial NF-κB activation in the (D; *n* = 3–5) small intestine and (E; *n* = 3–5) colon of LPS-injected BMCs, and (F) in small intestine and colon of *Myd88^−/−^* > p65^GFP-FL^x*Tlr4^−/−^*, *Ticam1^−/−^* > p65^GFP-FL^x*Tlr4^−/−^* BMCs. Each circle represents one mouse. Black line: median. Statistical analysis: Mann–Whitney *U* test. *, P ≤ 0.05; **, ≤ 0.01. Scale bars: 50 µm. Combined data of three (B), four (C), five (D), six (E), or seven (F) independent experiments. Each circle represents one mouse. Black line: median. Scale bars: 50 µm.

We injected the reporter mice i.v. with 5 µg ultrapure *Salmonella* Typhimurium (*S*. Tm) LPS, which activates TLR4, but no other relevant PRRs (see below). We chose this LPS concentration, which is ≥100-fold below the LD_50_ (Chen et al., 2015; Tateda et al., 1996), to model bacterial exposure during infection. We monitored epithelial NF-κB activation in the intestinal mucosa by two-photon microscopy imaging of intestinal explants. Of note, this assay might not be sensitive enough to observe low-level nuclear translocation of p65, so only full-blown NF-κB activation (concentration of p65^GFP-FL^ in the nucleus much higher than in the cytosol) is reliably detected with the presented setup. This is termed “NF-κB activation” hereafter, if not stated otherwise. We initially focused on cecal tissue, as this is the primary target site of the *S.* Tm infection model used later in this study, but also assessed gut segment specificity of the observed phenotypes. By 1 h postinjection (h.p.inj.) of LPS, ∼100% of the cecum IECs showed NF-κB activation (Fig. 1 A and Fig. S1 A). This coincided with the up-regulation of NF-κB target genes (*A20* [*Tnfaip3*], *Cxcl2*, and *Tnf*; Fig. S1 B). Due to the small nucleus size, the complex tissue architecture, and weak fluorescence of the p65^GFP-FL^ reporter, we could not resolve potential NF-κB activation in LP cells. As expected (Fitzgerald et al., 2003; Poltorak et al., 1998), NF-κB activation in our model depended on TLR4 and was absent in LPS-injected p65^GFP-FL^x*Tlr4^−/−^* mice (Fig. 1 A). To probe whether IECs are able to directly sense LPS via TLR4 in vivo, we generated bone marrow (BM) chimeras (BMCs) using p65^GFP-FL^ and p65^GFP-FL^x*Tlr4^−/−^* mice (Fig. 1 B). While p65^GFP-FL^ > p65^GFP-FL^x*Tlr4^−/−^* mice still showed ∼100% epithelial NF-κB activation in the cecum, p65^GFP-FL^x*Tlr4^−/−^* > p65^GFP-FL^ BMCs displayed NF-κB activation in only a fraction of IECs (Fig. 1 B). Accordingly, we detected stronger up-regulation of NF-κB target genes in the BMCs reconstituted with TLR4-proficient BM (Fig. S1 C). Small intestine and colon revealed a similar dependence on TLR4-proficient BM-derived cells (Fig. S1, D and E). Remaining radioresistant immune cells in tissues after irradiation represent a well-described confounding factor in studies using BMCs (Bogunovic et al., 2006). Based on the above, we concluded that the residual epithelial NF-κB activation observed in the p65^GFP-FL^x*Tlr4^−/−^* > p65^GFP-FL^ BMCs likely stemmed from residual radioresistant TLR4-proficient immune cells remaining in the recipients. To rigorously assess the role of TLR4 signaling by particular mucosal cell types, we therefore used p65^GFP-FL^x*Tlr4^−/−^* mice as recipients for the remaining BMC experiments. LPS injections into *MyD88^−/−^* > p65^GFP-FL^x*Tlr4^−/−^*, *Ticam1^−/−^* > p65^GFP-FL^x*Tlr4^−/−^*, and *MyD88^−/−^*x*Ticam1^−/−^* > p65^GFP-FL^x*Tlr4^−/−^* BMCs revealed MyD88 as the main downstream signal transducer in LPS-sensing immune cells of the cecum (Fig. 1 C) and small intestine (Fig. S1 F). Ticam1 induced only scattered foci of epithelial NF-κB activation in the absence of MyD88 (Fig. 1 C and Fig. S1 F). Previous work has shown that MyD88-mediated signaling induces an early, reliable, and transient NF-κB response, whereas Ticam1 signaling is more sensitive to cell-to-cell variation and leads to a later, prolonged response in some cells (Cheng et al., 2015). This higher sensitivity of Ticam1-mediated signaling to stochastic effects might explain the observed variability in epithelial NF-κB activation in *MyD88^−/−^* > p65^GFP-FL^x*Tlr4^−/−^* BMCs. By contrast, dependence of LPS-mediated NF-κB activation on robust but transient MyD88 signal transduction might contribute to restriction of signal propagation in cecum and small intestine (Cheng et al., 2015). Strikingly, MyD88 and Ticam1 appeared almost completely redundant in TLR4 downstream signaling in the colon (Fig. S1 F). This suggests a different prioritization of signal propagation versus containment in the colon compared with cecum and small intestine, which might be linked to increasing microbe exposure along the intestinal axis (Mowat and Agace, 2014).

**Figure S2.**
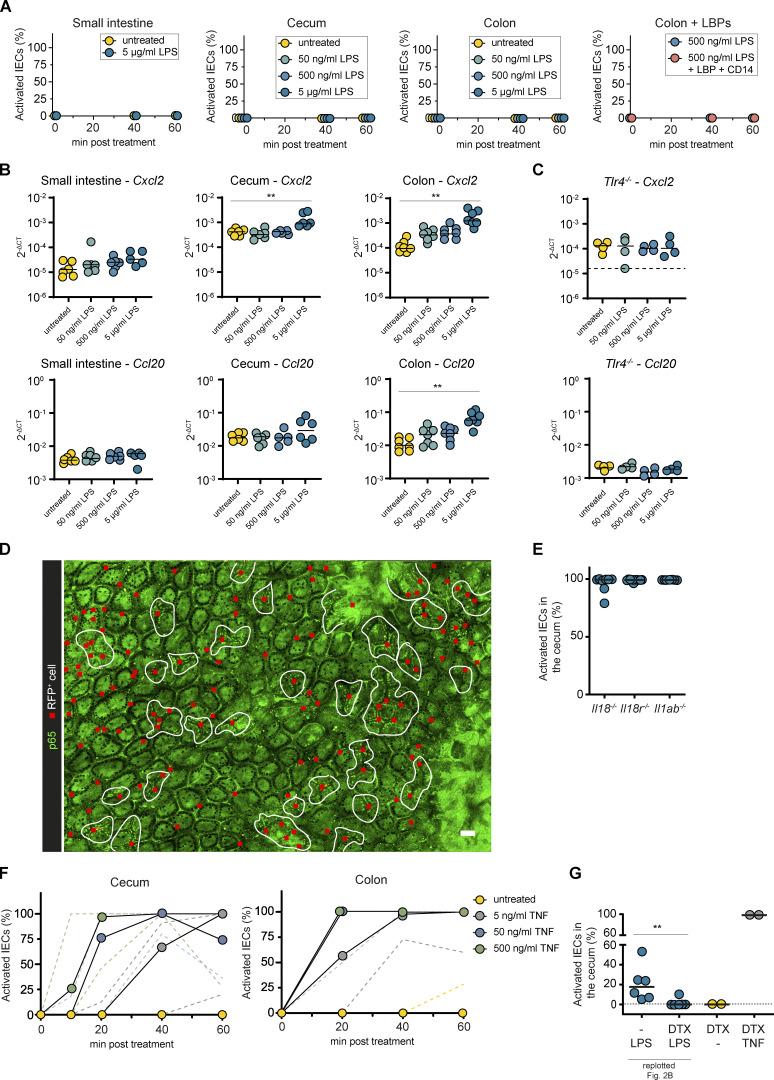
**TNF produced by CD11c^+^ cells induces local epithelial NF-κB activation in the intestinal mucosa. (A and B) **p65^GFP-FL^ intestinal epithelial organoids established from the indicated regions were treated with 5, 50, and 500 ng/ml or 5 µg/ml LPS (+ LBP and CD14, if indicated) and imaged for 1 h (A; *n* = 3–17), or analyzed by qPCR at 3 h of treatment (B and C; *n* = 6 or 7). **(C)** Colon organoids from p65^GFP-FL^x*Tlr4^−/−^* mice (*n* = 4). **(D)** Representative two-photon microscopy overview image of the cecal mucosa of mice described in Fig. 2 A at 1 h.p.inj. of LPS (*n* = 6). Red squares indicate RFP^+^ (*Tlr4^+/+^*) cells. White lines indicate IEC NF-κB activation zones (defined as areas with continuous epithelial NF-κB activation). **(E)** Quantification of epithelial NF-κB activation in *Il18^−/−^* > p65^GFP-FL^x*Tlr4^−/−^*, *Il18r^−/−^* > p65^GFP-FL^x*Tlr4^−/−^*, and *Il1ab^−/−^* > p65^GFP-FL^x*Tlr4^−/−^* BMCs at 1 h.p.inj. of LPS (*n* = 7 or 8). **(F)** p65^GFP-FL^ intestinal epithelial organoids from cecum (left) or colon (right) were treated with 5, 50, and 500 ng/ml TNF and imaged for 1 h (*n* = 3–17). **(G)** Quantification of epithelial NF-κB activation in mice as described in Fig. 2 B. Mice pretreated with DTX were injected with PBS or TNF (*n* = 2–6). Cecae were imaged at 1 h.p.inj. Data of LPS-injected mice are replotted from Fig. 2 B for comparison. Black line: median (B, C, E, and G). Dashed line: detection limit (C and G) or error range (A and F). Each circle represents one organoid sample (B and C), one mouse (E and G), or the median (A and F). Statistical analysis: one-way ANOVA with Dunett’s correction (B and C) or Mann–Whitney *U* test (E and G). *, P ≤ 0.05; **, P ≤ 0.01. Scale bars: 50 µm. Combined data of two (A, small intestine; B, C, D, and F, cecum), three (A, cecum), four (F, colon), six (B and E), or eight (A, colon) independent experiments.

To directly assess the responsiveness of IECs to LPS, we next generated intestinal epithelial organoids from the small intestine, cecum, and colon of p65^GFP-FL^ mice and treated them with LPS. The organoids did not respond with NF-κB activation to LPS stimulation (p65^GFP-FL^ imaging; Fig. 1 D and Fig. S2 A) independent of the presence of the TLR4 coreceptors LBP and CD14 (Frey et al., 1992; Lee et al., 1993; Wright et al., 1990). As previous reports showed low-level transcription of proinflammatory cytokines in colonic organoids upon LPS exposure (Kayisoglu et al., 2021; Price et al., 2018), we performed quantitative PCR (qPCR) on LPS-treated organoids. Accordingly, we observed a modest 2–10-fold increase of *Cxcl2* and/or *Ccl20* transcripts despite the absence of full-blown epithelial NF-κB activation (Fig. S2 B; compare Fig. S2 A). However, the level of induction was much lower than in the intestinal tissue of LPS-injected mice (>300-fold for *Cxcl2*; Fig. S1 B). Notably, this pertained to colonic, and partially cecal, but not to small intestinal organoids, and only occurred upon exposure to the highest concentrations of LPS tested (5 µg/ml). LPS treatment of *Tlr4^−/−^* colonic organoids confirmed TLR4 dependency of this response (Fig. S2 C). This indicates that, partially in line with previous reports (Kayisoglu et al., 2021; Price et al., 2018), organoids derived from different intestinal regions differ in LPS responsiveness. While small intestinal organoids are nonresponsive, cecal and colonic organoids can sense high concentrations of LPS in a TLR4-dependent manner. This sensing, however, does not induce full-blown NF-κB activation, and the transcriptional responses are modest in comparison to the whole tissue, suggesting that TLR4-induced NF-κB activation is specifically inhibited in these organoids (Allaire et al., 2021; Chassin et al., 2010; Lotz et al., 2006). As organoids robustly recapitulate primary IECs in vivo (Hausmann et al., 2020b; Kayisoglu et al., 2021), it is conceivable that similar regulating mechanisms occur in the murine colon. How TLR4 mediates this modest up-regulation of chemokine transcription independently of full-blown NF-κB activation in colonic organoids, and how the organoid differentiation status affects LPS responsiveness, remain to be assessed.

Taken together, these data indicate that primary IECs in the intact murine intestine do not directly respond with full-blown NF-κB activation to LPS via TLR4, but rather that NF-κB activation is induced by a secondary signal produced by radiosensitive immune cells.

### CD11c^+^ cells in the intestinal mucosa mediate locally restricted epithelial NF-κB activation via secretion of TNF

**Figure 2.**
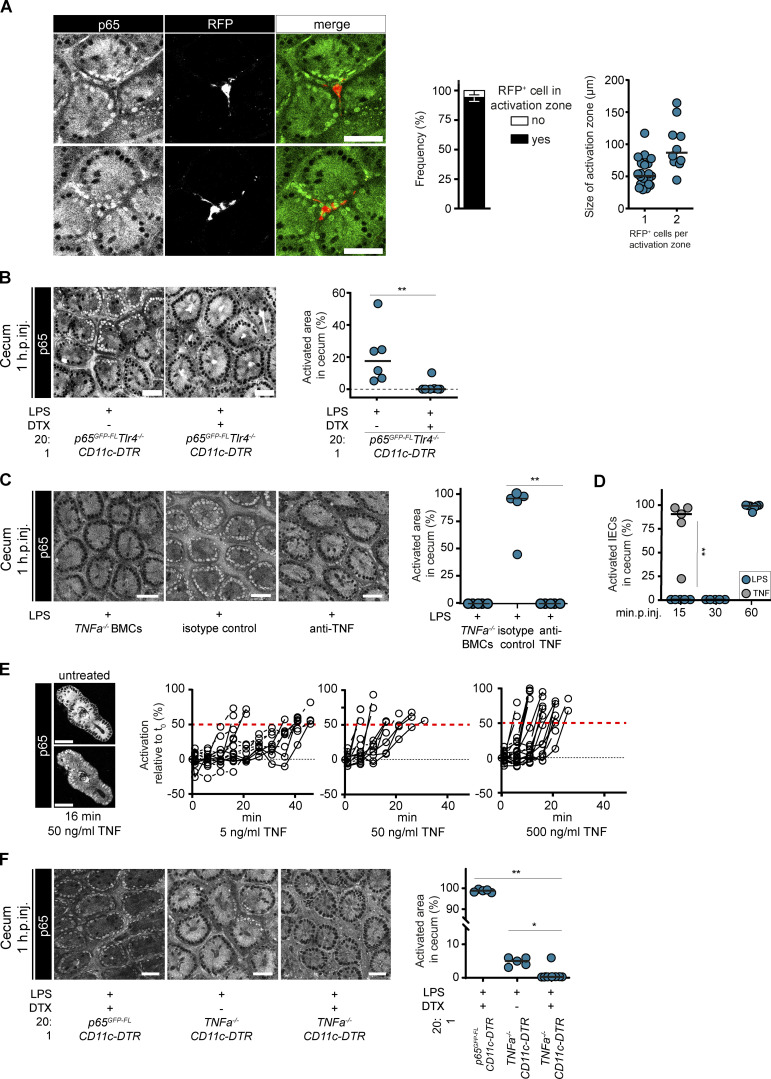
**CD11c^+^ cells induce local epithelial NF-κB activation via TNF.** Mice were i.v. injected with LPS and cecal explants imaged at 1 h.p.inj. by two-photon microscopy (representative image and quantification) if not indicated otherwise. **(A)** Cecum mucosa from p65^GFP-FL^x*Tlr4^−/−^* mice reconstituted with a 1:10 mix of *ActRFP* (10%, *Tlr4^+/+^*) and p65^GFP-FL^x*Tlr4^−/−^* (90%) BM. Analysis of RFP^+^ cells within an epithelial NF-κB activation zone (see Fig. S2 D, *n* = 10–18). **(B**–**D)** Cecal epithelium NF-κB activation of the indicated BMCs or p65^GFP-FL^ mice pretreated with isotype control/anti-TNF antibody or i.v. injected with TNF and analyzed at the indicated time points (*n* = 5 or 6). **(E)** TNF-treated small-intestinal epithelial organoids. Representative image and quantification of NF-κB activation kinetics with 5, 50, or 500 ng/ml TNF (*n* = 9–17). Lines connect data points from the same organoid. Red dashed line: 50% activation threshold. Black dotted line: no change. **(F)** Representative images of the cecal epithelium and quantification of epithelial NF-κB activation of p65^GFP-FL^x*Tlr4^−/−^* mice reconstituted with a 1:20 mix of CD11c-DTR and *TNFa^−/−^* BM, pretreated with DTX (*n* = 5–8). **(B–D and F)** Black line: median. *, P ≤ 0.05; **, P ≤ 0.01 by Mann–Whitney *U* test. Each circle represents one mouse or one organoid (E). Combined data of two (A and B), three (D and E), four (F), or six (C) independent experiments. Scale bars: 50 µm.

Our data point toward an indirect activation of epithelial NF-κB signaling via immune cells in the LP in vivo. To explore this further, we reconstituted irradiated p65^GFP-FL^x*Tlr4^−/−^* mice with a 1:10 mix of *ActRFP* (10%; *Tlr4^+/+^*) and p65^GFP-FL^x*Tlr4^−/−^* (90%) BM. This setup should allow us to observe signals emanating from individual RFP^+^ (*Tlr4^+/+^*) LP cells. Strikingly, LPS injection into these mixed BMCs resulted in epithelial NF-κB activation only close to RFP^+^, TLR4-proficient cells (Fig. 2 A and Fig. S2 D). Some RFP^+^ cells lacked an association with NF-κB–activated IECs, indicating that only a subset of these cells elicits the relevant signal by 1 h.p.inj. (verified below).

Interestingly, the area with continuous epithelial NF-κB activation (termed activation zone) around an RFP^+^ (*Tlr4^+/+^*) LP cell was rather small (median diameter, ∼50 µm; Fig. 2 A). This corresponds to the diameter of a crypt and indicates that the signal driving epithelial NF-κB activation must be locally confined. Local accumulation of RFP^+^ cells resulted in a larger activation zone (Fig. 2 A, right), pointing to a soluble signal. The shape of the RFP^+^ cells at the center suggested that they might be myeloid cells, a large fraction of which express MHCII and CD11c in the intestine (Tamoutounour et al., 2012). Myeloid cells are involved in induction of tissue responses after exposure to microbial stimuli (Arnold et al., 2016; Chikina et al., 2020; Corbin et al., 2020; Kinnebrew et al., 2012; Koscsó et al., 2020; Morita et al., 2019; Muzaki et al., 2016). To test this hypothesis, we reconstituted p65^GFP-FL^x*Tlr4^−/−^* mice with a 1:20 mix of *CD11c-DTR* (5%) and p65^GFP-FL^x*Tlr4^−/−^* (95%) BM. LPS injection into those BMCs resulted in ∼20% of IECs featuring NF-κB activation. Importantly, this activation was abolished by specific depletion of CD11c^+^ cells from the TLR4-proficient immune cell pool via diphtheria toxin (DTX; Fig. 2 B).

To identify the relevant signal, we probed the NF-κB–activating cytokines IL-1α, IL-1β, IL-18, and TNF (Collart et al., 1990; Hiscott et al., 1993; Kojima et al., 1999; Mori and Prager, 1996; Shakhov et al., 1990) via BMCs generated by reconstituting irradiated p65^GFP-FL^x*Tlr4^−/−^* mice with BM from the respective knockout mice. While mice reconstituted with *Il18^−/−^*, *Il18r^−/−^*, or *Il1ab^−/−^* BM still showed epithelial NF-κB activation after LPS injection (Fig. S2 E), reconstitution with *TNFa^−/−^* BM abolished this response (Fig. 2 C). The role of TNF was confirmed using TNF-neutralizing antibodies (Fig. 2 C) and was further supported by time course data. While epithelial NF-κB activation after LPS injection took ∼1 h, we observed epithelial activation as early as 15 min after TNF injection (Fig. 2 D). In organoids, TNF was sufficient for NF-κB activation within 15 min (Fig. 2 E, 50 ng/ml TNF; Fig. S2 F). Furthermore, TNF injection into the DTX-pretreated BMCs described in Fig. 2 B induced epithelial NF-κB activation (Fig. S2 G).

Finally, we verified that TNF was released directly by the LPS-sensing CD11c^+^ cells. We generated BMCs by reconstituting irradiated p65^GFP-FL^x*Tlr4^−/−^* mice with a 1:20 mix of *CD11c-DTR* (5%) and *TNFa^−/−^* (95%) BM. While LPS injection into those BMCs resulted in ∼5% epithelial NF-κB activation, the DTX depletion of CD11c^+^ cells from the pool of TNF-proficient cells completely abrogated epithelial NF-κB activation (Fig. 2 F). This established CD11c^+^ cells in the LP as the TNF-producing subset fueling full-blown epithelial NF-κB activation after LPS injection. Taken together, CD11c^+^ cell–derived TNF is both required and sufficient to drive NF-κB activation in IECs upon LPS exposure. This hints to a CD11c^+^ cell type as the missing link between the sentinel function of the mucosal immune system and epithelial NF-κB activation.

### LPS exposure induces a rapid response in LP cells, followed by secondary epithelial NF-κB activation

**Figure 3.**
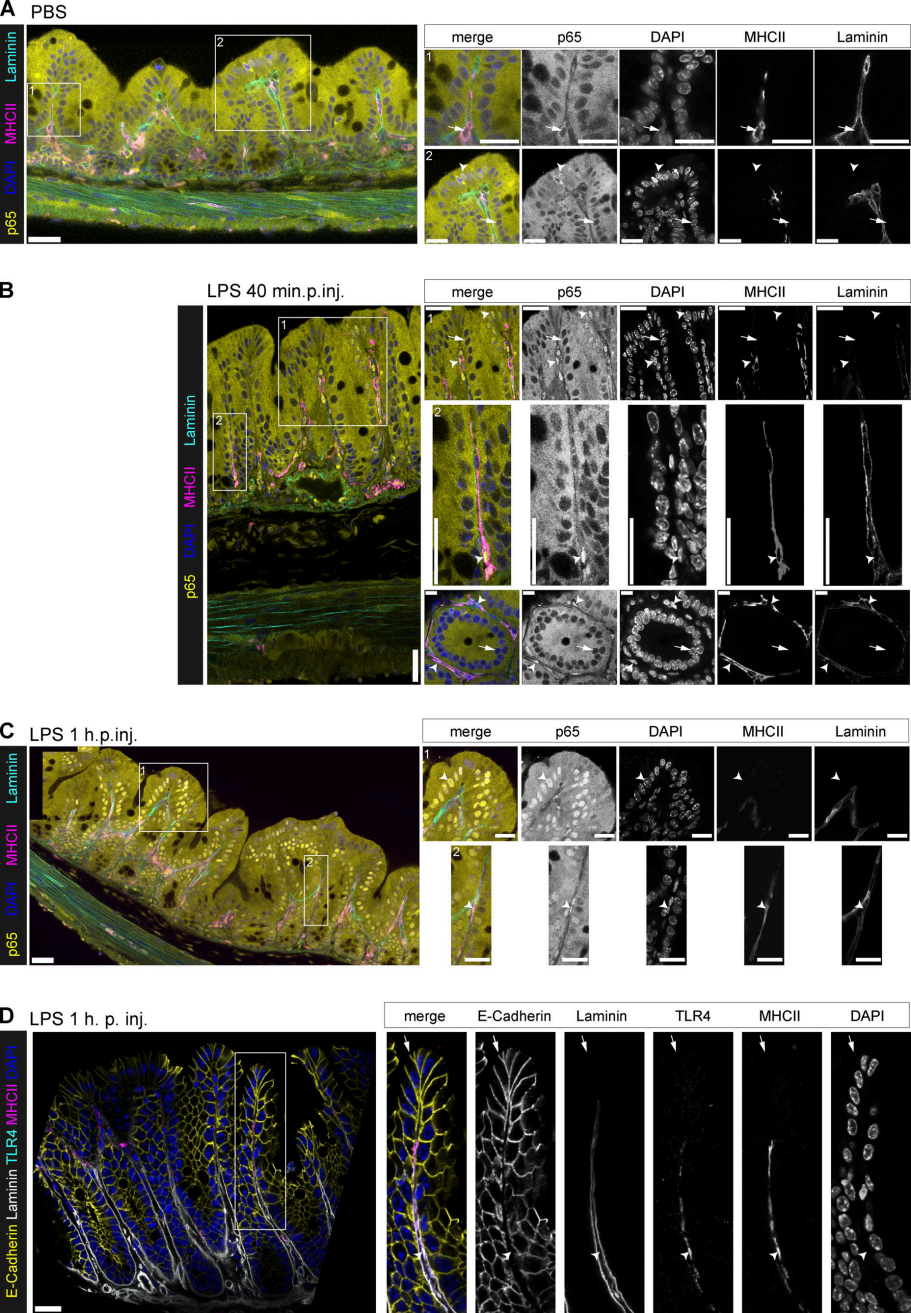
**LPS exposure induces a rapid response in LP cells, followed by secondary epithelial NF-κB activation. (A–D) **Confocal microscopy images of fixed cecae of p65^GFP-FL^ mice (A–C) or WT mice (D) i.v. injected with (A) PBS or (B–D) LPS and analyzed as indicated. Boxes in overview images indicate insets. Arrowheads indicate p65^+^ nuclei (A–C) or MHCII^+^ cells (D). Arrows indicate p65^−^ nuclei (A–C) or IECs (D). Scale bars: 30 µm (overview images A–C), 20 µm (overview image D), or 10 µm (insets A–C). Representative images of mice from three independent experiments (*n* = 4–7).

**Figure S3.**
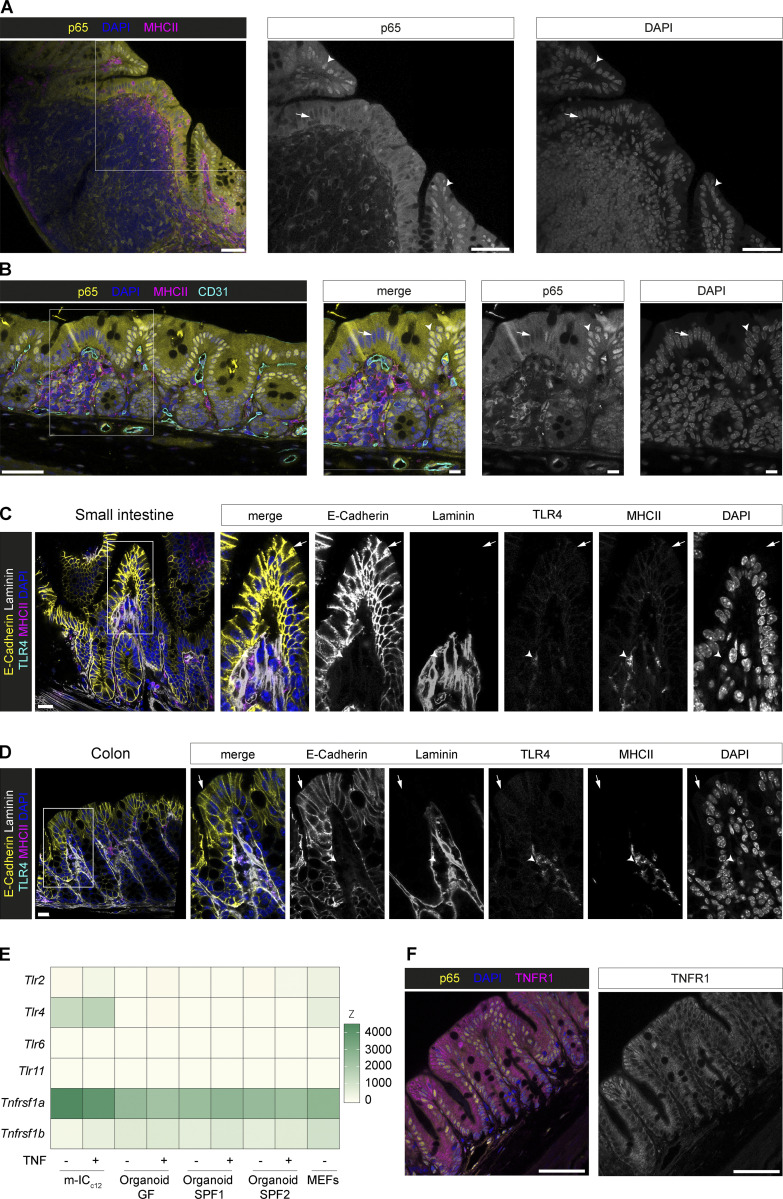
**Receptor expression in IECs. (A–D)** Confocal microscopy images of (A) the cecal patch, and (B) a mucosa-associated lymphoid follicle in fixed cecae of p65^GFP-FL^ mice i.v. injected with LPS at 1 h.p.inj. Boxes in overview images indicate insets. Arrowheads indicate p65^+^ nuclei. Arrows indicate p65^−^ nuclei. Scale bars: 50 µm (overview images) or 10 µm (insets). TLR4 staining in small intestine (C) and colon (D) of WT mice. Arrowheads indicate MHCII^+^ cells. Arrows indicate IECs. Scale bars: 20 µm. Representative images of mice from two experiments. **(E)** Heat map depicting expression levels of *Tlr2, Tlr4, Tlr6, Tlr11*, *Tnfrsf1a* (TNFR1), and *Tnfrsf1b* (TNFR2) in untreated or TNF-treated (5 ng/ml, 8 h) small intestinal epithelial organoids derived from SPF (SPF1, SPF2) or germ-free (GF) mice, m-IC_c12_ cells, and mouse embryonic fibroblasts (MEFs; reanalysis of a previously published transcriptome dataset, all detectable *Tlrs* depicted; Hausmann et al., 2020b). **(F)** Cecal mucosa stained for TNFR1 at 1 h.p.inj. of LPS. Scale bars: 50 µm. Representative images of mice from three independent experiments (*n* = 4–7).

To confirm the link to LPS-sensing LP cells and establish their localization within the mucosa, we applied a novel large-volume multi-color high-resolution fluorescence microscopy technique (Coutu et al., 2018; Kunz and Schroeder, 2019; Kokkaliaris et al., 2020) to fixed sections of naive and LPS-injected p65^GFP-FL^ mice. Again, we did not detect full-blown NF-κB activation in the bulk of IECs in naive mice (Fig. 3 A). Merely single apical IECs displayed low-level baseline NF-κB activation. At 40 min postinjection (min.p.inj.) of LPS, NF-κB activation was detectable in MHCII^+^ cells, which stretched out in the LP between crypts (Fig. 3 B). The CD11c^+^ TNF-producing LP cells identified above likely belong to this cell population. By contrast, full-blown epithelial NF-κB signaling occurred later (1 h.p.inj.; Fig. 3 C). In line with low baseline activation and subsequent desensitization, some apical IECs might fail to respond within 1 h (compare Fig. 3, A and C). Interestingly, our high-resolution imaging approach revealed that dome epithelium shielding the cecal patch and small lymphoid follicles specifically lacked NF-κB activation at 1 h.p.inj. (Fig. S3, A and B). In conclusion, these data support a temporally and physically spaced response to LPS, including early activation of CD11c^+^ MHCII^+^ LP cells and subsequent epithelial NF-κB activation. Notably, certain regionally differentiated IEC subpopulations displayed desynchronized NF-κB signaling kinetics.

LPS responsiveness may be limited by TLR4 expression. TLR4 expression in the small intestine is debated (Hornef et al., 2002; Kayisoglu et al., 2021; Lotz et al., 2006; Price et al., 2018), while several studies agree on TLR4 expression in the colon (Kayisoglu et al., 2021; Price et al., 2018; Wang et al., 2010). Using our high-resolution microscopy approach, we stained TLR4 on MHCII^+^ LP cells in cecum (Fig. 3 D), small intestine (Fig. S3 C), and colon (Fig. S3 D). Weaker/no TLR4 expression was detected in the epithelium (Fig. 3 D; and Fig. S3, C and D). Differential regulation of PRRs in an epithelial cell line compared with primary epithelial cells might explain some discrepancies in the literature (Hausmann et al., 2020b). Indeed, in contrast to other TLRs, *Tlr4* was highly expressed in m-IC_C12_ cells, an immortalized small IEC line previously used as a model for murine IECs (Bens et al., 1996; Hornef et al., 2002; Cario et al., 2000; Hornef et al., 2003), but virtually undetectable in small intestinal epithelial organoids (Fig. S3 E). Instead, IECs in vivo expressed high levels of TNFR1 (Fig. S3 F), as described before (Feng and Teitelbaum, 2013; Günther et al., 2015; Van Hauwermeiren et al., 2015; Storey et al., 2002) and in line with our transcriptome data (*Tnfrsf1a*; Fig. S3 E). In summary, these data support that epithelial NF-κB activation occurs indirectly in the LPS-exposed intestinal mucosa.

### TNF production upon LPS exposure in the intestinal mucosa is a local response driven by tissue-resident intercrypt macrophages

**Figure 4.**
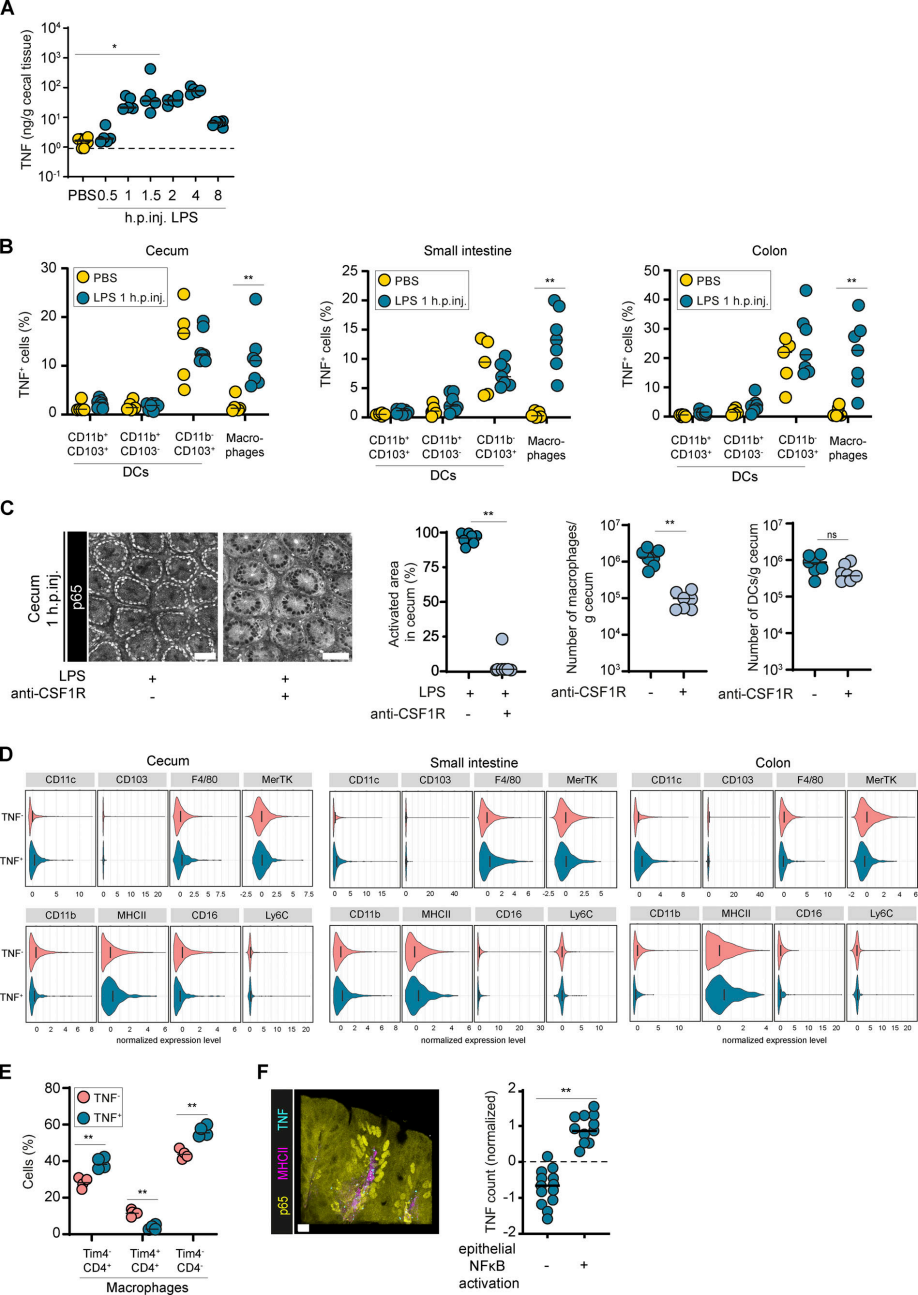
**Tissue resident, monocyte-derived macrophages secrete TNF to induce local epithelial NF-κB activation. (A)** ELISA measurements of TNF concentrations in the cecal mucosa of LPS injected WT mice (*n* = 5 or 6). Dashed line: detection limit. y axis in log_10_ scale. **(B)** Percentage of TNF^+^ DCs or macrophages (gating as shown in Fig. S4 D) in the cecum, small intestine, and colon of LPS-treated WT mice (1 h.p.inj.) and PBS-treated controls (*n* = 5–7). **(C)** Representative images of the cecal epithelium and quantification of epithelial NF-κB activation of p65^GFP-FL^ mice pretreated with anti-CSF1R or isotype control, injected with LPS, and imaged 1 h.p.inj. (*n* = 7). Depletion efficiency of macrophages and DCs in anti-CSF1R treated mice. **(D)** Normalized marker expression of TNF^+^ compared with TNF^−^ macrophages in the cecum, small intestine, and colon of LPS-injected WT mice (*n* = 4–7). **(E)** Percentage of CD4^+/−^ Tim4^+/−^ cells among TNF^−^ and TNF^+^ macrophages in the cecum of LPS-injected WT mice (*n* = 4). **(F)** TNF-PLA analysis of cecae from p65^GFP-FL^x*Tlr4^−/−^* mice reconstituted with a 1:40 mix of *ActRFP* (2.5%, *Tlr4^+/+^*) and p65^GFP-FL^x*Tlr4^−/−^* (97.5%) BM. Representative confocal microscopy image of fixed cecal tissue at 40 min.p.inj. (left) and quantification of PLA for TNF in crypts without (−) or with (+) epithelial NF-κB activation (Fig. S5 A) at 1 h.p.inj. (*n* = 11–13). Scale bar: 10 µm. Black line: median. Statistical analysis: one-way ANOVA with Dunett’s correction (A), two-way ANOVA with Sidak’s multiple comparison test (B), or Mann–Whitney *U* test (C, E, and F). *, P ≤ 0.05; **, P ≤ 0.01. Each circle represents one mouse (A–E) or one crypt (F; five mice analyzed). Combined data of two (D), three (B and C), four (F), or six (A) independent experiments, or exemplary data of two (E) independent experiments.

To verify TNF as a key driver of full-blown epithelial NF-κB activation, we measured TNF concentrations in cecal tissue of LPS-injected mice. TNF levels began to rise in the cecal mucosa by 0.5–1 h.p.inj., reaching a plateau of ∼50–100 ng/g tissue by 1.5–4 h.p.inj (Fig. 4 A). These kinetics correspond closely to the full-blown activation of epithelial NF-κB (compare with Figs. 2 and 3).

**Figure S4.**
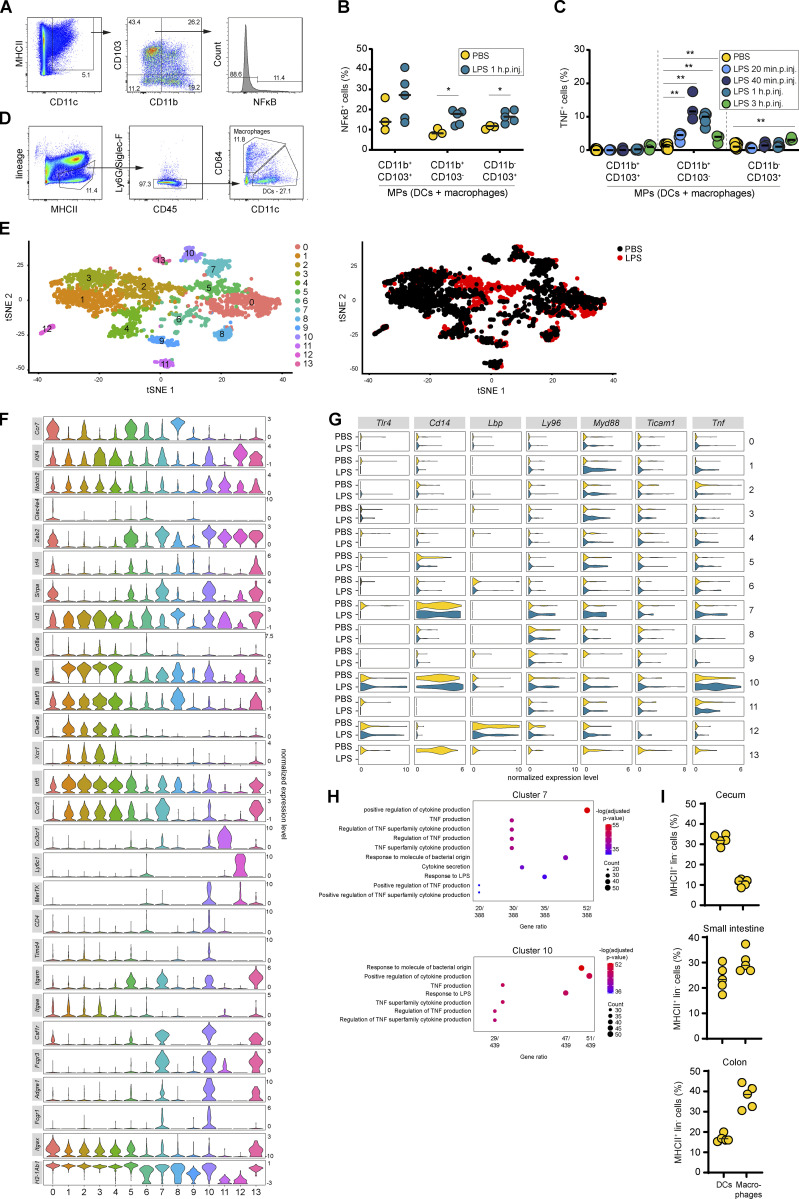
**Intestinal macrophages secrete TNF to induce local epithelial NF-κB activation. (A)** Gating strategy for intestinal MP subsets in the cecal mucosa of mice depicted in B and C. **(B and C) **Flow cytometry analysis of cecal MPs from PBS- or LPS-injected (B) KappaBle mice for assessment of NF-κB activation (gating as shown in A; *n* = 3–5) or (C) WT mice for identification of TNF-producing MP subsets (gating as shown in A; *n* = 3–6). **(D)** Updated gating strategy for differentiation of intestinal DCs and macrophages as shown in Fig. 4, B, D, and E. Lineage = NK1.1, CD3, B220. **(E)** For scRNAseq, CD45^+^ live MHCII^+^ lineage (NK1.1, CD3, B220)^−^ cells were sorted from the cecal mucosa of 40 min LPS-injected mice or PBS-treated controls (*n* = 4 mice) and subsequently analyzed by scRNAseq (10X Genomics). T-distributed stochastic neighbor embedding plots showing the distribution of the analyzed cells indicated by cluster (left) or treatment (right). **(F)** Expression levels of intestinal MP markers: this analysis revealed two clear macrophages clusters (7 and 10). CD11b^−^ CD103^+^ Xcr1^+^ DCs were represented in clusters 1–4, out of which cluster 2 mainly consisted of cells from LPS-treated mice, indicating that this might represent an activated state. This is in line with the secondary TNF production of this subset at later time points after injection, as detected by flow cytometry (C). Clusters 0 and 5 were positive for *Sirpa* and contained CD11b^+^ CD103^+^ DCs (cluster 0) and CD11b^+^ CD103^−^ DCs (cluster 5). While clusters 12 and 13 were positive for a number of monocyte/macrophage markers and therefore likely represent maturing macrophages, the assignment of clusters 6, 8, and 9 was challenging due to overlapping marker expression. These clusters, together with cluster 11 (mast cells), likely contained precursors (macrophage/DC, cluster 8) or contaminating cells (T cells, cluster 6 and 8; plasma cells, cluster 9). **(G)** Expression analysis of TLR4 signaling–associated genes. **(H)** Gene set enrichment analysis for macrophage clusters (7 and 10). **(I)** Frequency of DCs and macrophages in the cecum, small intestine, and colon of naive WT mice (*n* = 5). Each circle represents one mouse (B, C, and I) or one cell (E). Black line: median. Combined data of two (I), three (B), or nine (C) independent experiments. Statistical analysis: one-way ANOVA with Tukey’s correction (C) or Mann–Whitney *U* test (B). *, P ≤ 0.05; **, P ≤ 0.01.

To identify the TNF-producing CD11c^+^ cell type, we analyzed intestinal myeloid cell populations by flow cytometry using KappaBle mice, which express destabilized GFP under a synthetic NF-κB–controlled promoter (Tortola et al., 2021 *Preprint*). Upon LPS injection, both MCHII^+^ CD11c^+^ CD11b^+^ CD103^−^ and CD11b^−^ CD103^+^ mononuclear phagocyte (MP) subsets expressed GFP (Fig. S4, A and B). Intracellular TNF was specifically identified in the CD11b^+^ CD103^−^ MP subset (Fig. S4 C). Again, the TNF production peaked at ∼40 min.p.inj., while the fraction of TNF producing CD11b^+^ CD103^−^ MPs declined by 3 h.p.inj. (Fig. S4 C). The CD11b^−^ CD103^+^ intestinal MP subset also began producing TNF, though at much lower levels and no earlier than 3 h.p.inj. This latter TNF production is likely attributable to secondary activation, and its kinetics suggest that it is not the driver of epithelial NF-κB activation by 1 h.p.inj. Instead, CD11b^+^ CD103^−^ intestinal MPs constitute the relevant source of TNF, explaining this swift epithelial NF-κB activation in their immediate vicinity.

To further define the TNF-producing cell type, we extended our staining panel (Fig. S4 D; Joeris et al., 2017; Tamoutounour et al., 2012). This identified a macrophage subpopulation, which is present in cecum, small intestine, and colon (Fig. 4 B). In line with that, macrophage depletion by anti-CSF1R treatment (Arnold et al., 2016) abrogated LPS-induced epithelial NF-κB activation (Fig. 4 C). The relevant TNF-producing cells are CD11c^hi^, F4/80^hi^, MerTK^hi^, MHCII^hi^, CD16^lo^, CD4^+/−^, Tim4^−^, tissue-resident macrophages (Fig. 4, D and E; Joeris et al., 2017). CD4 and Tim4 define three intestinal macrophage subpopulations with distinct turnover kinetics: CD4^+^ Tim4^+^ macrophages maintain locally, independent of blood-derived monocytes. CD4^+^ Tim4^−^ macrophages have a slow turnover, while CD4^−^ Tim4^−^ macrophages are rapidly replenished. The two latter subpopulations derive from blood monocyte precursors (Bain et al., 2013; Shaw et al., 2018). The main fraction of TNF-producing macrophages is CD4^−^ Tim4^−^, indicating a fast turnover of this population. This is well in line with the efficient depletion of the TNF-producing population by irradiation in the BMCs. The smaller fraction of low-turnover CD4^+^ Tim4^−^ sentinel macrophages could represent the remaining TNF-producing cells in the BMC experiments (Fig. 1 B).

Single-cell RNA sequencing (scRNAseq) of cecal MP populations (Fig. S4, E and F) from PBS- and LPS-injected mice revealed high expression of the TLR4 signaling module (*Tlr4*, *Cd14*, *Lbp*, *Ly96* [MD2], *Myd88*, *Ticam1*) in the macrophage clusters (7 and 10; Fig. S4 G). Interestingly, *Tnf* mRNA was abundant in cecal macrophages even at baseline (Fig. S4 G). TNF production in macrophages can be regulated posttranscriptionally to prepare for quick responses upon activation (Han et al., 1990; Kontoyiannis et al., 1999). Gene set enrichment analysis revealed a strong TNF signature in the macrophage clusters (Fig. S4 H), further supporting this hypothesis. The low frequency of macrophages among cecal MPs compared with small intestine and colon (Fig. S4 I) and the transient nature of the response described here did not offer enough resolution for comparing the TNF-producing subset with previously identified macrophage subsets in other parts of the intestine (Chikina et al., 2020; Corbin et al., 2020; Kang et al., 2020). Nonetheless, the presented data are consistent with our observations described above and show that macrophage subsets can express the relevant (co)receptors for LPS sensing.

Moreover, and in line with the late response to LPS exposure shown in Fig. S4 C, a large subpopulation of CD11b^−^ CD103^+^ dendritic cells (DCs) was present mainly in LPS-treated mice (cluster 2), indicating that they represent an activated subset. This CD11b^−^ CD103^+^ DC subset also expressed higher baseline *Tnf* levels compared with other DC populations, which is in line with the high baseline TNF production observed in these cells by flow cytometry (Fig. 4 B).

**Figure S5.**
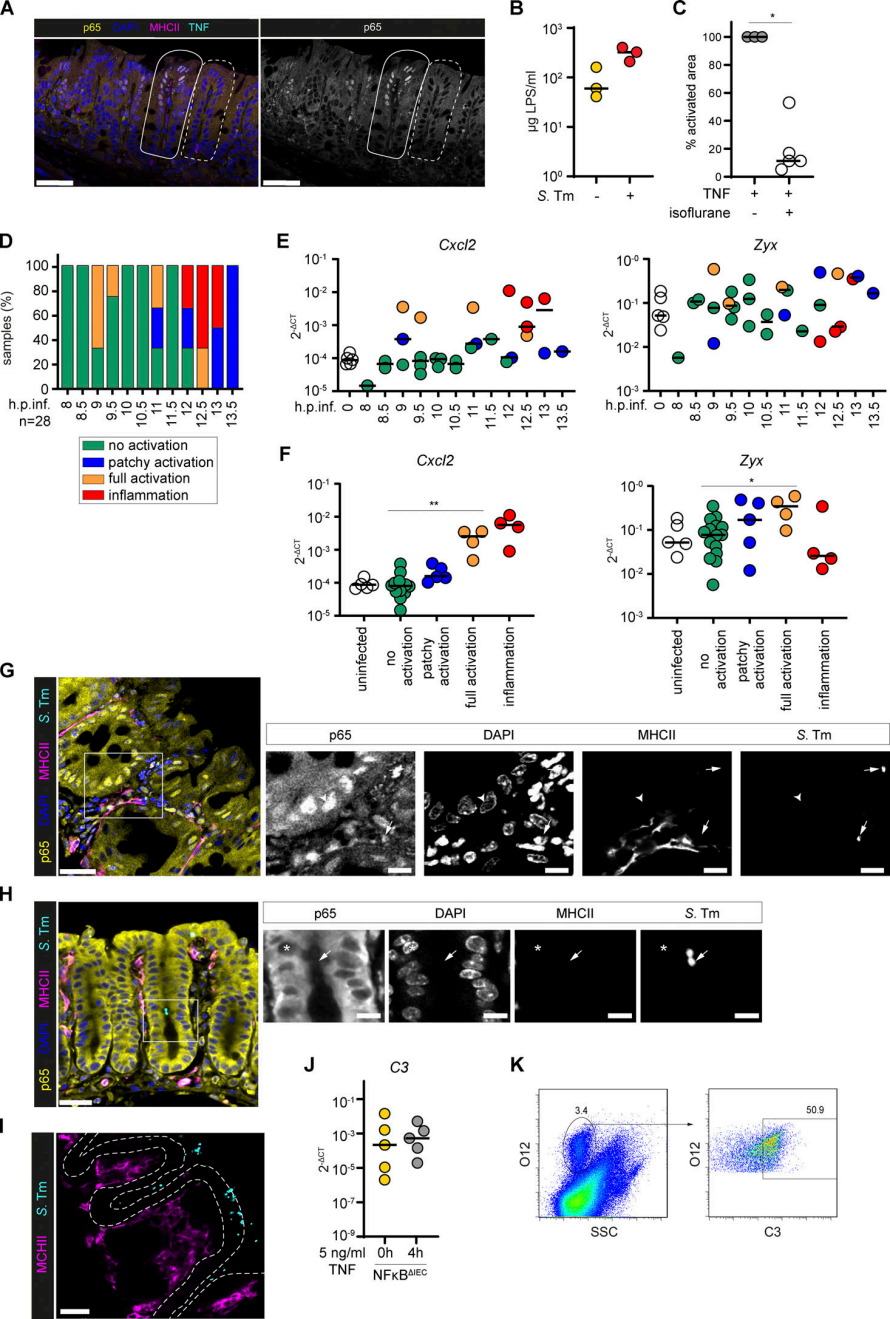
**Intestinal epithelial NF-κB activation status of *S*. Tm–infected mice correlates with mucosal expression of NF-κB target genes.** **(A)** Representative image of TNF-PLA in the cecal mucosa of LPS-injected mice as described in Fig. 4 F. Dashed line indicates a crypt without epithelial NF-κB activation. Solid line indicates a crypt with epithelial NF-κB activation. Scale bars: 50 µm. **(B)** LPS concentrations in the cecum lumen of untreated mice and mice that were streptomycin-pretreated and infected with *S*. Tm for 24 h (*n* = 3). **(C)** Quantification of epithelial NF-κB activation in the cecum of TNF-treated mice with or without isoflurane anesthesia at 15 min.p.inj. (*n* = 3–5). **(D)** Relative distribution of intestinal epithelial NF-κB activation status of *S*. Tm–infected mice described in Fig. 5 A, sorted by time of infection (*n* = 28). **(E and F)** Transcript levels of *Cxcl2* and *Zyx* in the cecal mucosa of mice described in A and naive p65^GFP-FL^ mice, grouped according to (E) the time point of infection (color code as in A) or (F) the epithelial NF-κB activation status of the respective mice. Expression levels were normalized to *Actb* and depicted in 2^-ΔCT^ (*n* = 33). **(G–I)** Confocal microscopy images of mice infected with *S*. Tm for 12 h (*n* = 6). 3D visualizations in Video 1, Video 2, and Video 3. Boxes in overview images indicate insets. Arrowheads indicate p65^+^ nuclei (G). Asterisks indicate p65^−^ nuclei (H). Arrows indicate *S*. Tm in LP (G) or in the lumen (H). Dashed line indicates the epithelium (I). Scale bars: 30 µm (overview images) or 10 µm (insets). **(J)** *C3* transcription levels in untreated and TNF-treated (5 ng/ml, 4 h) NF-κB^ΔIEC^ small intestinal epithelial organoids depicted as 2^-ΔCT^. Expression levels were normalized to *Actb* (*n* = 5). **(K)** Gating strategy for analysis of C3-coated *S*. Tm in the intestinal lumen (Fig. 5 G). Combined data of two (B and C) or five (J) independent experiments or representative images of five independent experiments (G–I). Statistical analysis: Mann-Whitney *U* test. *, P ≤ 0.05; **, P ≤ 0.01. Black line: median. Each circle represents one mouse (B–F) or one experiment (average, J).

To visualize the macrophage–IEC crosstalk in the mucosa, we applied high-resolution microscopy (Kunz and Schroeder, 2019) to mixed BMCs. Similar to Fig. 2 A, we reconstituted irradiated p65^GFP-FL^x*Tlr4^−/−^* mice with a 1:40 mix of *ActRFP* (2.5%, *Tlr4^+/+^*) and p65^GFP-FL^x*Tlr4^−/−^* (97.5%) BM and performed a proximity ligation assay (PLA) for TNF on fixed cecal tissue after LPS injection. This setup allowed us to use crypts without RFP^+^ cells in the LP (i.e., in which epithelial NF-κB activation was not triggered; Fig. S5 A, dashed line) as internal, on*-*slide controls. This revealed a stronger TNF signal specifically within and in immediate vicinity of MHCII^+^ LP cells localized in crypts with epithelial NF-κB activation (Fig. 4 F). In summary, our combined data demonstrate that intercrypt macrophages secrete TNF to trigger locally restricted epithelial NF-κB activation in the intestinal mucosa upon LPS exposure.

### TNF-mediated NF-κB activation in IECs occurs upon bacterial infection and induces antibacterial epithelial responses

Our injection protocol exposes the basolateral side of the gut epithelium to LPS, while natural infections would initially present LPS at the apical side. To relate the amount of injected LPS to physiological LPS concentrations present in the intestine, we measured LPS concentrations in cecal content of naive and *S*. Tm–infected mice. The luminal LPS concentration of naive mice was ∼100-fold higher than that of the injected LPS (assuming a homogenous distribution; Fig. S5 B). As naive mice lack full-blown epithelial NF-κB activation, this supports that the intact intestinal epithelium is largely unresponsive to luminal microbe–derived LPS.

**Figure 5.**
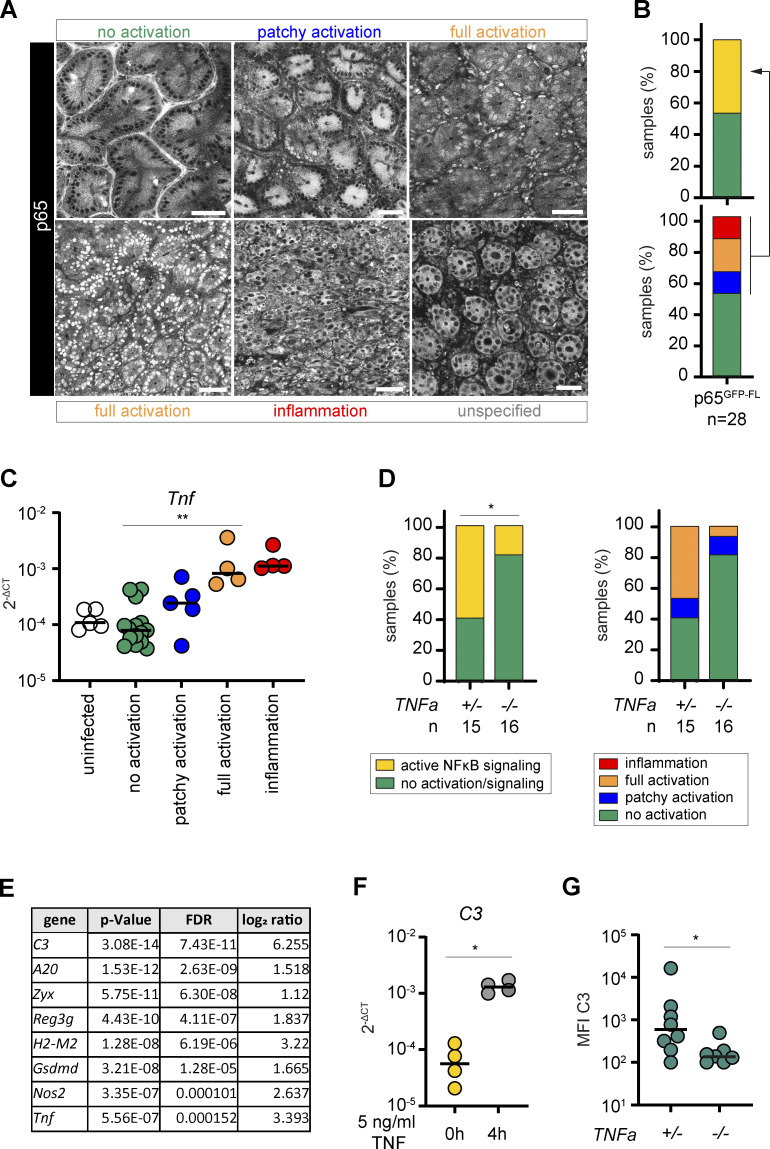
**TNF-mediated epithelial NF-κB activation occurs upon bacterial infection and induces an antibacterial response. (A)** Representative two-photon microscopy images of cecal explants of streptomycin-pretreated p65^GFP-FL^ mice infected with *S*. Tm for 8–13.5 h (*n* = 28). Categories for scoring of epithelial NF-κB activation status: “no activation” (green); “patchy activation” (blue); “full activation” (orange); “inflammation” (red; tissue distortion evident); “unspecified” (gray; was excluded from further analysis). Scale bars: 50 µm. **(B)** Distribution of the analyzed 28 samples of A among the four epithelial NF-κB activation categories (bottom). For simplification, the blue, orange, and red categories were summarized as “NF-κB signaling” (yellow, top). **(C)** *Tnf* transcript levels in the cecal mucosa of mice described in A and naive p65^GFP-FL^ mice, grouped according to the epithelial NF-κB activation status of the respective mice and depicted as 2^-ΔCT^. Expression levels were normalized to *Actb* (*n* = 33). **(D)** *TNFa^+/−^* or *TNFa^−/−^* > p65^GFP-FL^x*Tlr4^−/−^* BMCs were analyzed as described in A and B. **(E)** Log_2_ ratios of selected genes in a transcriptome analysis of TNF-treated (8 h, 5 ng/ml) compared with untreated small intestinal epithelial organoids (Hausmann et al., 2020b). FDR, false discovery rate. **(F)** *C3* transcript levels in untreated and TNF-treated (5 ng/ml, 4 h) small intestinal organoids depicted as 2^-ΔCT^. Expression levels were normalized to *Actb*. **(G)** Streptomycin-pretreated *TNFa^−/−^* mice and heterozygous littermates were orally infected with *S*. Tm for 36h. *S*. Tm in the cecal lumen (gated on O12^+^ cells, see Fig. S5 K) were stained for surface C3 to assess coating of luminal bacteria by flow cytometry (C3^+^ population). MFI, median fluorescence intensity. Statistical analysis: Mann–Whitney *U* test (C, F, and G) or χ^2^ test (D). *, P ≤ 0.05; **, P ≤ 0.01. Each circle represents one mouse (C and G) or one experiment (average; F). y axis in log_10_ scale (C, F, and G). Combined data of three (G), four (F), five (A–C), or six (D) independent experiments.

By contrast, infection with the invasive Gram-negative bacterium *S*. Tm activated epithelial NF-κB to a similar degree as LPS injections. In streptomycin-pretreated WT (here p65^GFP-FL^) mice, *S*. Tm invades the cecum epithelium and thereby elicits a pronounced, acute inflammatory response by ∼8–12 h postinfection (h.p.inf.; Barthel et al., 2003). As large areas of the cecal epithelium lack a protective mucus layer (Furter et al., 2019), the disease pathology is limited to the cecal mucosa at this initial phase of the infection. We first attempted to analyze epithelial NF-κB responses during infection using a previously established in vivo real-time microscopy approach (Müller et al., 2012). The isoflurane anesthesia, however, inhibited the onset of NF-κB signaling (Fig. S5 C). To avoid artifacts, we therefore used end point analysis of cecal explants (as above), which does not require isoflurane. We harvested cecum tissue from *S*. Tm–infected mice at 8–13.5 h.p.inf. and performed two-photon microscopy. While epithelial NF-κB activation was detectable in those mice, the degree of activation varied considerably between the animals, with no direct correlation to the time of infection (Fig. 5 A and Fig. S5 D). This was attributable to inter-individual differences in the infection kinetics, to which noise-sensitive Ticam1 signaling might also contribute (compare with Fig. 1 C; Cheng et al., 2015). To stratify the samples with respect to their effective state in the infection process, we sorted them based on the extent of epithelial NF-κB signaling from “no activation” (green), via “patchy activation” (blue) and “full activation” (orange), to “inflammation” (red; tissue distortion evident; Fig. 5 A and Fig. S5 D). In a subgroup of samples, we did not detect p65^+^ epithelial nuclei, but they clearly differed from the samples categorized as “no activation.” This sample group (“unspecified,” gray) most likely represents a state of active epithelial transcription (enlarged nuclei) and onset of inflammation in the tissue (space between crypts enlarged, edema). However, we have not analyzed this in detail and therefore excluded this sample group from further analysis.

NF-κB target genes were up-regulated in correlation to the tissue NF-κB activation status (Fig. S5 E; compare with Fig. S5 F). Calculating the ratio of samples with NF-κB activation out of all analyzed samples at 8–13.5 h.p.inf., ∼50% of the mice showed either partial or tissue-wide epithelial NF-κB signaling (Fig. 5 B, yellow). Importantly, the epithelial NF-κB activation status significantly correlated with *Tnf* expression levels in the cecal tissue, hinting toward a role of TNF in this phenotype (Fig. 5 C).

To probe the role of TNF in epithelial NF-κB signaling during the infection, we generated BMCs by reconstituting p65^GFP-FL^x*Tlr4^−/−^* mice with either *TNFa^+/−^* or *TNFa^−/−^* BM. In *TNFa^+/−^* > p65^GFP-FL^x*Tlr4^−/−^* BMCs analyzed at 8–13 h.p.inf., a similar fraction of samples featured epithelial NF-κB signaling as in our previous experiments (Fig. 5 D, compare Fig. 5 B). By contrast, *TNFa^−/−^* > p65^GFP-FL^x*Tlr4^−/−^* BMCs featured significantly fewer animals with active epithelial NF-κB signaling (Fig. 5 D). Taken together, these data show that also during early *S*. Tm infection, TNF production by radiosensitive immune cells contributes significantly to epithelial NF-κB activation.

The relevance of this pathway was further supported by imaging data (Fig. S5 G and Video 1). Notably, and in line with the insensitivity of IECs to LPS, engagement of *S*. Tm with the luminal side of the epithelium was not sufficient to activate NF-κB signaling in these crypts (Fig. S5 H and Video 2). This indicates that IECs serve as an LPS-inert barrier to prevent sustained mucosal activation by luminal LPS (Fig. S5 I and Video 3).

**Video 1.** **Epithelial activation zone with underlying MHCII^+^ cell–associated *S*. Tm.** 3D visualization of the image data of the tissue that was sampled to generate Fig. S5 G. Rendered *S*. Tm were filtered for MHCII-associated *S*. Tm (see Materials and methods). Arrows indicate MHCII+ cell–associated *S. *Tm. 24 frames per second.

**Video 2.** **Epithelium-associated *S*. Tm does not trigger epithelial NF-κB activation.** 3D visualization of the image data of the tissue that was sampled to generate Fig. S5 H. 24 frames per second.

**Video 3.** **The intestinal epithelium shields luminal bacteria from intercrypt sentinel macrophages.** 3D visualization of the image data of the tissue that was sampled to generate Fig. S5 I. 24 frames per second.

A previously published organoid transcriptome dataset was reanalyzed to decipher TNF-induced gene expression programs and identify candidate defenses (Hausmann et al., 2020b). In organoids, TNF exposure induced well-characterized NF-κB target genes, including several host defense effectors (Fig. 5 E; Hausmann et al., 2020b; Leppkes et al., 2014). The complement component *C3* (∼40–50-fold up-regulation; Fig. 5, E and F) was induced in an NF-κB–dependent manner (Fig. S5 J). C3 production by IECs is supported by previous reports (Matsumoto et al., 2017; Sina et al., 2018; Sünderhauf et al., 2017). To test how the absence of TNF would affect C3 production during *S*. Tm infection, we infected *TNFa^−/−^* mice and heterozygous littermate controls with *S*. Tm. At 36 h.p.inf., we stained the gut luminal *S*. Tm for C3 surface coating and measured the levels by flow cytometry. C3 coating of gut luminal *S*. Tm was significantly reduced in *TNFa^−/−^* mice (Fig. 5 G and Fig. S5 K), although still detectable. This indicates that TNF is important, but not absolutely required, for eliciting the production of pathogen-coating C3 in the mucosa.

Taken together, TNF promotes epithelial NF-κB signaling not only upon LPS injection but also during oral *S*. Tm infection. Importantly, the extent of epithelial NF-κB activation in the mucosa correlates with the induction of a number of prominent antibacterial defense mechanisms. An array of those IEC-produced factors (here exemplified by C3) is induced by TNF. This highlights how a parallelly wired network drives antibacterial responses in the intestine, and partially explains why it has been challenging to pinpoint the contribution of individual genes and cell types in the defense against bacterial infection (Abeler-Dörner et al., 2020). Despite these challenges, we have here identified a linear multi-component circuit in vivo, whereby TLR4-MyD88(/Ticam1) in intercrypt sentinel macrophages senses bacterial LPS, resulting in the release of TNF, which drives attuned local epithelial NF-κB signaling.

## Discussion

TLR4 is well-established as the receptor for noncytosolic LPS (Poltorak et al., 1998). TLR4 on myeloid cells induces proinflammatory responses upon LPS binding (Beutler and Rietschel, 2003; Medzhitov and Janeway, 2000b; Shakhov et al., 1990). Previous work, largely based on epithelial cell lines, also indicated a role for IEC TLR4 in sensing LPS (Cario et al., 2000; Hornef et al., 2002, 2003). A recent study using fluorescent reporter mouse lines described TLR4 expression in the colon and, at low levels, in the small intestine (Price et al., 2018). Technical obstacles (Hausmann et al., 2020b; Price et al., 2018), the complex regulation of TLR4 reactivity (Chassin et al., 2010; Negishi et al., 2006; Zhang et al., 2006), regional differences (Kayisoglu et al., 2021; Price et al., 2018; Wang et al., 2010), and the dynamic expression of TLR4 during development and disease (Cario and Podolsky, 2000; Dheer et al., 2016; Lotz et al., 2006) have, however, made it difficult to pinpoint functional relevance of epithelial TLR4 in the intestine. Our analysis shows that LPS itself cannot directly elicit full-blown NF-κB activation in IECs in vivo. Of note, LPS concentrations of 5 µg/ml induce modest transcription of known NF-κB target genes (*Cxcl2*, *Ccl20*) in organoids from the colon (and to a lesser extent from the cecum) independently of full-blown NF-κB activation. Whether this type of activation occurs in vivo and if it significantly contributes to physiological responses remain unclear. Integrating previous studies with our data, we conclude that TLR4 expression in homeostatic IECs is at best low. This pertains especially to the cecum and small intestine, whereas expression of TLR4 by colonic IECs is reported by several studies from different laboratories (Günther et al., 2015; Kayisoglu et al., 2021; Price et al., 2018; Wang et al., 2010). The dependence on microbiota differences (Wang et al., 2010) might explain why we do not detect IEC TLR4 expression in our model. In line with our observations, however, a number of studies report no functional evidence of IEC TLR4 in their models despite detecting TLR4 expression in colonic IECs (Günther et al., 2015; Price et al., 2018). This indicates that TLR4 expression and functional relevance are not necessarily congruent.

In line with the above and earlier work (Günther et al., 2015), we found a regionally consistent dependence of IEC NF-κB activation after LPS treatment on immune cell TLR4 in cecum, small intestine, and colon. This applies to unperturbed adult mice with a relatively controlled microbiota. We cannot refute a role of epithelial TLR4 in settings of chronic inflammation, or during development (Cario and Podolsky, 2000; Lotz et al., 2006). The control of IEC responses to LPS in vivo by lack of TLR4 or coreceptor expression (Kayisoglu et al., 2021), or by inhibition of epithelial TLR downstream signaling (Chassin et al., 2010; Lotz et al., 2006), remains to be fully resolved. We can, however, conclude that IEC TLR4 has no functional relevance as an LPS sensor for induction of full-blown NF-κB activation in vivo in the mature, unperturbed mouse gut.

We identified monocyte-derived tissue-resident macrophages located in intercrypt regions of the LP to elicit epithelial NF-κB activation by secreting TNF upon LPS exposure. This activation is spatially restricted. The dependence of signal transduction on MyD88 (Cheng et al., 2015) as well as the control of macrophage activation by IL-10 (Girard-Madoux et al., 2016; Zigmond et al., 2014) might contribute to this restriction by limiting the duration of the macrophage response. One intercrypt macrophage activates an epithelial zone of ∼50 µm in the adjacent crypts. In line with their role as tissue sentinels, intercrypt macrophages stretch out through the entire length of the LP between crypts. Upon LPS exposure, these intercrypt macrophages react within 40 min with NF-κB activation and TNF production, which in a next step elicits NF-κB signaling in IECs. scRNAseq data confirmed high transcription of the components of the TLR4/MD2/CD14/LBP-MyD88/Ticam1-TNF axis specifically in the macrophage subsets, which is in apparent contrast to previous reports on anergy of intestinal macrophages (Bain et al., 2013; Mowat et al., 2017; Schridde et al., 2017; Smythies et al., 2005). High baseline transcription of *Tnf* could point to posttranscriptional regulation of TNF production in these macrophages (Han et al., 1990; Kontoyiannis et al., 1999) and might represent a mechanism to allow a swift response upon activation. Taken together, these data support that a tight regulation of proinflammatory signaling (Girard-Madoux et al., 2016; Schridde et al., 2017; Smith et al., 2011; Zigmond et al., 2014) rather than lack of PRR expression (Smythies et al., 2005) controls the homeostatic phenotype of intestinal macrophages. This is in line with derailed macrophage responses in chronic intestinal inflammation (Arnold et al., 2016; Corbin et al., 2020; Bain et al., 2013).

The data presented here show that the epithelium forms an LPS-unresponsive physical barrier that limits bacterial tissue translocation. Intercrypt macrophages, in turn, act as sentinels for invasive Gram-negative bacteria, and secrete TNF as first responders to induce a locally restricted response, likely across different IEC subsets. While DC–IEC crosstalk is well established (Kinnebrew et al., 2012; Muzaki et al., 2016), evidence for macrophage–IEC crosstalk consolidated recently in different contexts (Bernshtein et al., 2019; Chikina et al., 2020; Morhardt et al., 2019; Serena et al., 2019). The localization and shape of the sentinel intercrypt macrophages identified here suggest that they might belong to a recently described CD11c^+^ CD121b^+^ CD206^int^ macrophage subset (Kang et al., 2020). Future work should verify this.

Notably, TNF mediated induction of epithelial NF-κB activation represents a crypt-scale response, triggering a parallelized antibacterial program in IECs. This includes several central IEC chemokines and antibacterial effectors (here exemplified by C3). Our data highlight the redundant wiring of tissue responses to pathogen insults, which ensures proficient defense even if individual effector mechanisms are lacking or corrupted by the pathogen. Such redundancy may in part explain the difficulty of pinpointing individual contributions of single genes and/or cell types to pathogen defense and likely also in the context of IBD (Abeler-Dörner et al., 2020).

Taken together, we have identified monocyte-derived tissue-resident intercrypt macrophages as first responders to exposure to bacterial LPS. They act as sentinels in the LP that rapidly detect bacterial LPS via TLR4 and secrete TNF to induce a local epithelial NF-κB–mediated antibacterial program. Notably, the spatially restricted nature of this communication ensures triggering of an antibacterial response only in the close vicinity of the microbial insult. The signaling circuit identified here therefore represents a tunable defense mechanism to induce appropriate responses according to the localization and intensity of a microbial trigger, similar to the concept of immune response regulation via quorum sensing that was recently proposed (Bardou et al., 2021; Postat et al., 2018). We suggest that this spatial organization may help to prevent overshooting immune activation at the tissue scale and thereby exacerbation of tissue inflammation.

## Materials and methods

### Mouse experiments

All animal experiments were performed in accordance with legal and ethical regulations. Experiments were approved by Kantonales Veterinäramt Zürich (licenses 222/2013, 193/2016, and 158/2019). The mice were housed in individually ventilated cages under specific pathogen–free conditions at the Eidgenössische Technische Hochschule (ETH) Phenomics Center or Rodent Center HCI at ETH Zürich. All transgenic animals presented here have a C57BL/6 background. With the exception of the BMCs, mice were 8–12 wk old at the time of experimentation. Cohoused heterozygous littermates were used as controls where applicable (Fig. 5, D and G). The following mouse lines were used: C57BL/6J (WT; *Ly5.2*, in-house breeding), *Ly5.1* (B6.SJL-*Ptprca Pepcb/*BoyJ; Charbonneau et al., 1988; backcrossed for >10 generations), p65^GFP-FL^ (De Lorenzi et al., 2009), p65^GFP-FL^x*Tlr4^−/−^* (this study, generated by crossing p65^GFP-FL^ mice with B6.129-*Tlr4^tm1Aki/Aki^* mice; Hoshino et al., 1999), *ActRFP* (B6.Cg-Tg[CAG-DsRed*MST]1Nagy/J; Vintersten et al., 2004; backcrossed for >10 generations), *VillinRFP* (Müller et al., 2012; backcrossed for >5 generations), KappaBle (Tortola et al., 2021 *Preprint*), *Il1ab^−/−^* (B6.D-*Il1a^tm1Yiw^*/*Il1b^tm1Yiw^*; Horai et al., 1998), *TNFa^−/−^* (B6.129-*Tnf^tm1Ljo^*; Marino et al., 1997; backcrossed for >10 generations), *MyD88^−/−^*x*Ticam1^−/−^* (this study, generated by crossing B6.129-*Myd88^tm1Aki^*; Adachi et al., 1998; backcrossed for >20 generations with B6.B6-*Ticam1^LPS2^*/J [Hoebe et al., 2003] mice), NF-κB^ΔIEC^ (*Rela*^fl/fl^ *Relb*^fl/fl^ *c-Rel*^fl/fl^ *Villin-Cre*^tg/WT^, backcrossed for >10 generations; Vlantis et al., 2016), and *CD11c-DTR* (B6.FVB-*1700016L21Rik^Tg(Itgax-DTR/EGFP)57Lan^*/J; Jung et al., 2002; backcrossed for >5 generations). For LPS/TNF treatment, mice were i.v. injected with 5 µg ultrapure *S*. Tm LPS (kind gift of Otto Holst, Research Center Borstel Borstel, Germany) or 3 µg TNF (Preprotech) in 100 µl PBS and euthanized at the indicated time points after injection. For DTX treatment, mice were i.p. injected with 120 ng DTX (Sigma-Aldrich) at 24 h before LPS injection. For anti-TNF treatment, mice were i.p. injected with 200 µg anti-TNF antibody (InVivoMAb; BE0058) or the respective isotype control (InVivoMAb; BE0088) in 100 µl PBS 24 h before LPS injection. For anti-CSF1R treatment, the mice were i.v. injected with 1 mg anti-CSF1R antibody (InVivoMAb; BE0213 [AFS98]) or the respective isotype control (InVivoMAb; BE0088 [2A3]) at 4 d before LPS injection, and subsequently 0.3 mg i.v. at 3–1 d before LPS injection. For isoflurane treatment, mice were kept under isoflurane anesthesia (1.5–3%) for 15 min after TNF injection. For *S*. Tm infection, mice were pretreated with 25 mg streptomycin by intragastrical gavage 24 h before infection as described previously (Barthel et al., 2003). *S*. Tm SB300 (Hoiseth and Stocker, 1981; carrying no plasmid or pZ400 [SPI2^mCherry^]) was grown for 12 h at 37°C shaking in LB/0.3 M NaCl supplemented with 50 µg/ml streptomycin, diluted 1:20 and sub-cultured for 4 h before infection. Mice were infected with 5 × 10^7^ bacteria by intragastrical gavage and euthanized at the indicated time points.

### Generation of BMCs

For BMC experiments, recipient mice were irradiated (950 rad) at 6–12 wk of age. Donor BM was isolated, washed in 20 ml ice-cold PBS (BioConcept), and resuspended at a concentration of 10^7^ cells/ml. Recipients were i.v. injected with 5 × 10^6^ donor BM cells. For mixed BMCs, donor cells were counted after isolation and mixed in the respective ratios before injection. Mice were given Borgal (Veterinaria AG) in the drinking water for 3 wk and used for experiments after 6–20 wk of reconstitution.

### Organoid culture and treatment

Intestinal epithelial organoids were established as described before. The incubation time of the tissue pieces in gentle dissociation reagent was extended to 20 min for the establishment of cecum and colon organoids. Organoids were cultured for 3–4 d after splitting before TNF treatment, and sampled as described previously (Hausmann et al., 2020b). The following organoid cultures were used: WT (small intestine): Z908, Z911, AG120; NF-κB^ΔIEC^: AE118; p65^GFP-FL^ (small intestine): W35, AB268; p65^GFP-FL^ (cecum): AO105; p65^GFP-FL^ (colon): AM51; p65^GFP-FL^x*Tlr4^−/−^* (colon): AO557.

### Two-photon microscopy

For explant microscopy, mice were i.v. injected with LPS or TNF, or infected with *S*. Tm as described above. At the indicated time points, mice were euthanized. The intestine was excised and cut open longitudinally, and the intestinal content was carefully removed. The intestinal mucosa was mounted onto a slide (Thermo Fisher Scientific), submerged in DMEM F12 medium (Life Technologies), and subsequently imaged. For organoid imaging, organoids were seeded in 10 µl Matrigel domes into 8-well chambers on a microscopy slide (Thermo Fisher Scientific) or into 96-well plates (Greiner Bio-One; 655090). Imaging was performed on a Leica SP8 DMI 6000B microscope equipped with an HC PL IRAPO CORR 40×/1.10 water immersion objective, using filters for GFP (525/50) and RFP (585/40) and Leica HyD SP GaAsP detectors, located at ScopeM, ETH Zürich. Excitation was performed with a Mai Tai XF Laser (Spectra-Physics) tuned to 920 nm, and an InSight DeepSee Laser (Spectra-Physics) tuned to 1,110 nm. Image acquisition and data extraction were performed with the Leica application suite 3. For measuring of epithelial NF-κB activation, all epithelial nuclei were enumerated according to the p65-GFP signal (counting epithelial cells with and without evidence for nuclear NF-κB activation). Then we counted the epithelial cells with p65^+^ nuclei to calculate the percentage of p65^+^ epithelial nuclei (Fig. 1, A–D; Fig. 2, D and E; and Fig. S2, A and E–G). Alternatively, the total area as well as the area with epithelial NF-κB activation were measured to calculate the percentage of activated area (Fig. 2, B, C, and F; Fig. 4 C; and Fig. S1, D–F). Image analysis was performed with Fiji 1.51n.

### Confocal microscopy

For high-resolution fluorescence microscopy (Fig. 3; Fig. S3, A–D and F; and Fig. S5 A), samples were fixed in 4% PFA for 4 h at 4°C. Subsequently, the cecal content was flushed out manually. Samples were washed in ice-cold PBS for 3 × 2 min and stored in PBS at 4°C until further processing. Staining was conducted using primary and secondary antibodies as described (Coutu et al., 2018). Briefly, cecum samples were embedded in low-gelling temperature agarose and cut into 150-µm-thick cross-sections using a vibratome. The tissue sections were permeabilized using TBS (plus 0.05% Tween and 1% Triton X) and blocked with 10% donkey serum. Subsequently, primary and secondary antibodies were applied overnight and for 3 h, respectively. Tissue sections were mounted in homemade mounting medium (80% glycerol, and 20% TBS containing 0.1 M N-propyl gallate, pH 8.5) in silicon molds, to avoid sample compression, on 1.5 coverslips. Image acquisition was performed on a Leica SP8 microscope, using the Leica 63× Glycerol Objective with Leica Glycerol immersion medium. The antibodies used for the stainings are listed in Tables 1 and 2.

**Table 1.** Primary antibodies

Antigen	Manufacturer	Clone/product ID
MHCII	Biolegend	110002
TLR4	BioRad	MCA2154T
GFP	Novus Biologicals	NB600-308
CD31	R&D Systems	AF3628
TNFR1	R&D Systems	AF-425
TNF	R&D Systems	AF-410
LamininAF647	Novus Biologicals	NB300-144AF647

**Table 2.** Secondary antibodies, dyes, and visualization kits

Antibody	Manufacturer	Clone/product ID
Donkey anti-rat Cy3	Jackson ImmunoResearch	712-165-153
Donkey anti-rabbit AF488	Thermo Fisher Scientific	A-21206
Donkey anti-goat AF488	Thermo Fisher Scientific	A-11055
Donkey anti-rat biotin	Jackson ImmunoResearch	712-065-153
Donkey anti-goat biotin	Jackson ImmunoResearch	705-065-147
Streptavidin 555	Thermo Fisher Scientific	S21381
Streptavidin 633	Thermo Fisher Scientific	S21375
Donkey anti-goat MINUS	Merck	DUO92006
Donkey anti-goat PLUS	Merck	DUO92003
PLA Detection Kit Far Red	Merck	DUO92013-100RXN
DAPI	Thermo Fisher Scientific	D1306

For three-dimensional (3D) immunofluorescence microscopy (Fig. S5 G–I; Video 1; Video 2; and Video 3), the samples were processed and stained with primary and secondary antibodies as described previously (Oderbolz et al., 2021) with integration of a nonheating microwave (Radtke et al., 2020). Briefly, cecum samples were embedded in 4% agarose and subsequently cut into 200-µm-thick tissue sections using a Compresstome (Precisionary). The tissue sections were permeabilized using 0.1% PBS/Tween20 and blocked with 10% goat serum. For microwave-assisted staining, a PELCO BioWave Pro+ (Ted Pella, Inc.) was used with an alternance of 2 min at 100 W and 1 min at 0 W. A 15-cycles program was executed for primary antibody labeling (Table 3) and a 10-cycles program for secondary antibody labeling (Table 4). Finally, the samples were washed in PBS overnight and mounted in a homemade refractive index matching solution with refractive index = 1.47 and supplemented with 0.1 M N-propyl gallate.

**Table 3.** Primary antibodies

Antigen	Manufacturer	Clone	Product ID
GFP	Novus Biologicals	NB600-308	Polyclonal
I-A/I-E	BioLegend	107601	M5/114.15.2
*S*. Tm O12	A. Lanzavecchia	N/A	hSTA5

**Table 4.** Secondary antibodies

Antibody	Fluorophore	Manufacturer	Clone
Goat, anti-rabbit	AF488	Abcam	Ab150077
Goat, anti-rat	AF555	Thermo Fisher Scientific	A-21434
Goat, anti-human	AF647	Jackson ImmunoResearch	109-605-098

Images of 200-µm-thick tissue sections were acquired with an inverted confocal microscope (Zeiss LSM 980 Airyscan) using 25× and 40× magnification objectives (numerical aperture of, respectively, 0.8 and 1.2; immersion: glycerin). Images were acquired with sequential fluorophore excitation, z-stack sizes of 0.5 µm, and a scan format of 512 × 512 pixels. Image analysis was performed using ImageJ/Fiji software (Version 1.51) and Imaris 9.5 (Bitplane). For visualization purposes, the gamma of the MHCII and *S*. Tm channels was adjusted to 0.75, and a median filter was used. The shortest distances from *S*. Tm surfaces to MHCII surfaces were computed (Table 5), and positive values were filtered out for visualization of MHCII^+^ cell–associated *S*. Tm only.

**Table 5.** Surface creation parameters for Video 1, Video 2, and Video 3

Surface/ spots name	Source channel	Smoothing: surface details (μm)	Background subtraction: diameter of largest sphere (μm)	Absolute intensity threshold (μm)	Split touching objects: split seed diameter (μm)	Quality threshold	Voxel number threshold
MHCII surfaces	MHCII AF555	0.5	15	8.3	10	6.3	1,500
*S*. Tm surfaces	*S*. Tm. AF647	0.5	-	4.3	-	-	-

### PLA

The PLA was performed as described before (Kunz and Schroeder, 2019). Briefly, cecum samples were cut and permeabilized as described above. Primary antibodies were added overnight. Instead of fluorescently labeled secondary antibodies, for TNF, PLA-secondary antibodies, recognizing the constant region of the goat-IgG of the anti-TNF antibody, were added. Further fluorescently labeled secondary antibodies were added for 3 h. After overnight incubation of the PLA secondary antibodies, the ligation and rolling circle amplification were performed on the slide at 37°C. After the PLA, the tissue sections were mounted and imaged as described above.

For the quantification of the TNF PLA signal per crypt (epithelial NF-κB activation versus no activation), we segmented the MCHII^+^ signal coarsely and separated it in distinct crypts based on the tissue morphology. We then counted the TNF signals within each MHCII isosurface (corresponding to a single activated or nonactivated crypt), yielding TNF counts per volume. To normalize for differences in staining efficiency between different slides, we performed z-transformation on the TNF counts per sample (slide).

### ELISA measurements

For ELISA measurements, small pieces of tissue were snap-frozen and kept at −80°C until further analysis. For organoid samples, two wells were pooled to obtain one sample. Cecal tissue was weighed before analysis, homogenized in 300 µl washing buffer of the ELISA kit, and spun down for 5 min at 4°C. The supernatant was used directly or diluted for the ELISA, which was performed according to the manufacturer’s instructions (TNF-ELISA: Invitrogen; BMS607HS).

### LPS measurement in cecal content

Cecal content was collected in 500 μl LPS-free water, homogenized by bead-beating (25/s), and stored overnight at −20°C. The cecal content was weighed before analysis and diluted, and the assay was performed according to the manufacturer's instructions (Lonza PyroGene Recombinant Factor C Endpoint Fluorescent Assay; 50-648U).

### LP cell isolation and flow-cytometric analysis

Cecum LP cell isolation and staining were performed as described previously (Hausmann et al., 2020a). For the analysis of small-intestinal (ileum) and colonic (proximal colon) LP cells, 3 or 2 cm of the respective part of the intestine were used, following the same protocol as for cecum LP cell isolation. For intracellular TNF staining, cell isolation and staining was performed in the presence of 5 µg/ml Brefeldin A (Biologend). After surface staining, cells were fixed and permeabilized in 100 µl PermMix solution (BD Biosciences red blood cell lysis buffer, diluted 1:5 in double distilled H_2_O, 1:1,000 Tween20) for 10 min at room temperature. After washing, the cells were incubated with anti-TNF antibody for 30 min at room temperature, washed, and resuspended in FACS buffer (1% heat-inactivated FCS and 5 mM EDTA in PBS) for subsequent flow-cytometric analysis. For the extended flow cytometry panel, cells were incubated in 1 µg/sample Mouse BD Fc Block (BD Biosciences) in 75 µl 10% Brilliant stain buffer (BD Biosciences)/FACS buffer for 5 min at room temperature before surface staining (this step was omitted when CD16-BUV395 was included in the staining panel). For surface staining, 25 µl of antibody mix in 10% Brilliant stain buffer/FACS buffer was subsequently added. A *TNFa*^−/−^ mouse served as control for the definition of the TNF^+^ gate. The following antibodies/reagents were used for the staining: CD45-PerCP (Biolegend; 30-F11; 1:100), CD45-BUV573 (BD Biosciences; 30-F11; 1:100), MHCII-APC (Biolegend; M5/114.15.2; 1:400), MHCII-BV421 (Biolegend; M5/114.15.2; 1:100), CD103-PE (Biolegend; 2E7; 1:100), CD11b-BV605 (Biolegend; M1/70; 1:200), CD11c-PE/Cy7 (Biolegend; N418; 1:200), CD3-BV711 (Biolegend; 145-2C11; 1:200), NK1.1-BV711 (Biolegend; PK136; 1:200), B220-BV711 (Biolegend; RA3-6B2; 1:200), Ly6G-APC/Cy7 (Biolegend; 1A8; 1:100), Siglec-F-APC/Cy7 (Biolegend; E50-2440; 1:200), CD64-AF647 (Biolegend; X54-5/7.1; 1:200), F4/80-BV785 (Biolegend; BM8; 1:200), CD16-BUV395 (BD Biosciences; 2.4G2; 1:100), Tim4-PerCP-Cy5.5 (Biolegend; RMT4-54; 1:100), CD4-BV785 (Biolegend; RM4-5; 1:100), Ly6C-AF700 (Biolegend; HK1.4; 1:200), MerTK-SB645 (Life Technologies; DS5MMER; 1:100), TNF-FITC (Biolegend; MP6-XT22; 1:100), Sytox-blue (Invitrogen; 1:1,000), Zombie NIR Fixable Viability Kit (Biolegend; 1:1,000), and LIVE/DEAD Fixable Aqua Dead Cell Stain (Life Technologies; 1:1,000). Samples were measured on a LSRII (BD Biosciences) or LSR Fortessa (BD Biosciences), and data were analyzed with FlowJo V10 (TreeStar).

### scRNAseq

Cecal MPs of WT mice injected with PBS or LPS (40 min.p.inj.) were isolated as described above and sorted for CD45^+^ live MHCII^+^ lineage^−^ cells as described previously (Hausmann et al., 2020a). The cells of four mice per treatment group were pooled for sorting.

Single-cell sequencing was performed at the Functional Genomics Center Zurich. The cell lysis and RNA capture were performed according to the 10X Genomics protocol (Single Cell 3′ v3 chemistry). The cDNA libraries were generated according to the manufacturer’s protocol (Illumina) and further sequenced (paired-end) with NovaSeq 6000 technology (Illumina). The transcripts were mapped with the 10Xgenomics CellRanger pipeline (version 4.0.0). The count matrices were analyzed with Seurat package v4.0 (Hao et al., 2020) in R 3.6.0 or 4.0.0 (R Core Team, 2006) using default parameters unless stated otherwise. Briefly, the count matrices were filtered (genes detected in <10 cells and cells with <700 transcripts were removed). Cells having >10% transcripts encoding mitochondrial genes were filtered out. Outlier cells (based on the correlation between total unique molecular identifiers and number of detected genes) were also removed. Clusters of cells displaying B cell, T cell, or mast cell phenotypes were excluded as well. The matrices were normalized (to 10,000 transcripts per cell), logged, and scaled per gene (mean 0 and variance 1). The resulting matrix was used to select the top 2,000 variable genes, which were used to compute principal components. The first 20 principal components were used for graph-based clustering (Louvain modularity; resolution parameter = 0.5). The first 20 principal components were also used for dimensionality reduction (t-distributed stochastic neighbor embedding; van der Maaten and Hinton, 2008). Next, differential expression analysis (log2 fold change > 0.2) was performed for all detected clusters (using Wilcoxon rank-sum test with multiple test correction; Benjamini and Hochberg, 1995). Gene set enrichment analysis was done using the GSEA package (Subramanian et al., 2005) and gene ontology database (Ashburner et al., 2000) on the differentially up-regulated genes (adjusted P value < 0.05) in each cluster. The count matrices were deposited in the European Nucleotide Archive database (accession no. PRJEB46461).

### Bacterial flow-cytometric analysis

For the assessment of C3 coating of *S*. Tm in the cecal lumen, cecal content was homogenized. The bacteria were fixed in 2% PFA/PBS for 20 min at room temperature, washed in PBS, and incubated with rat anti-C3 (Abcam; 11H9; 1:200), rabbit anti-O5 (Difco; Antiserum; 1:200), and human anti-O12 (kind gift from Antonio Lanzavecchia, Institute for Research in Biomedicine, Università della Svizzera Italiana, Bellinzona, Switzerland; STA5; 1:500) antibody in 1% BSA/PBS for 30 min at room temperature. After washing with 1% BSA/PBS, bacteria were incubated in anti-rat–FITC (1:200), anti-rabbit–BV421 (1:200), and anti-human–Alexa 647 (1:200) antibody in 1% BSA/PBS for 40 min at 4°C. Subsequently, bacteria were washed, resuspended in PBS, and analyzed on a CytoflexS cytometer (Beckmann Coulter). C3^+^ bacteria were gated according to a fluorescence minus one control.

### Gene expression analysis

Tissue RNA extraction (Hausmann et al., 2020a), organoid RNA extraction (Hausmann et al., 2020b), and qPCR analysis of the respective genes were performed as previously described (Hausmann et al., 2020a).

### Bulk transcriptome analysis

The transcriptome dataset described previously (Hausmann et al., 2020b) was partially reanalyzed with R Studio (version 3.6.0).

### Statistical analysis

Statistical analysis was performed with GraphPad Prism 8 or R Studio (version 3.6.0).

### Online supplementary information

Fig. S1 supplements Fig. 1 and shows gene transcription of NF-κB target genes in PBS and LPS treated animals, as well as NF-κB activation in the small intestine and colon of *Tlr4*^−/−^, *MyD88*^−/−^, and *Ticam1*^−/−^ BMCs. Fig. S2 supplements Fig. 2 and shows NF-κB activation and gene transcription in LPS- and TNF- treated organoids, as well as an overview of activation zones around RFP^+^ cells and NF-κB activation in TNF injected DTX-depleted mixed BMCs. Fig. S3 supplements Fig. 3 and shows NF-κB activation in dome epithelium, TLR4 expression in small intestine and colon, and TNFR1 expression in the cecum. Fig. S4 supplements Fig. 4 and shows the gating strategies used in Fig. 4, the percentages of NF-κB^+^ or TNF^+^ MPs in the cecum, and scRNAseq data on cecal MPs in PBS- and LPS-injected mice. Fig. S5 supplements Figs. 4 and 5 and shows crypts with or without NF-κB activation used for quantification in Fig. 4 F. Furthermore, it shows the distribution of the epithelial NF-κB activation states according to the time point of infection, as well as gene transcription levels sorted by time point after infection or by NF-κB activation status. Finally, it shows *S*. Tm within or in association with the cecal mucosa in the presence or absence of epithelial NF-κB activation, as well as *C3* gene expression in TNF-treated organoids and the gating strategy used to define C3^+^ bacteria for Fig. 5 G. Video 1 is a 3D visualization of the tissue shown in Fig. S5 G. Video 2 is a 3D visualization of the tissue shown in Fig. S5 H. Video 3 is a 3D visualization of the tissue shown in Fig. S5 I.
